# Towards Sustainable Development Goals: Application of Hydrogen‐Enriched Mahua Biodiesel/Diesel Blend to Dual‐Fuel Diesel Engine

**DOI:** 10.1002/gch2.202500260

**Published:** 2025-09-13

**Authors:** Anh Tuan Hoang, Swarup Kumar Nayak, Milan Vujanović, M. Olga Guerrero‐Pérez, Enrique Rodríguez‐Castellón, María Cruz López‐Escalante, Shams Forruque Ahmed, Hady Hadiyanto, Van Chinh Luu, Van Nhanh Nguyen, Xuan Phuong Nguyen, Dao Nam Cao

**Affiliations:** ^1^ Energy‐Fuel Technology and Applied Material Research Group Dong Nai Technology University Dong Nai Vietnam; ^2^ Faculty of Engineering Dong Nai Technology University Dong Nai Vietnam; ^3^ Graduate School of Energy and Environment Korea University 145 Anam‐ro, Seongbuk‐gu Seoul 02841 South Korea; ^4^ School of Mechanical Engineering & School of Chemical Engineering Kalinga Institute of Industrial Technology (Deemed to be University) Odisha 751024 India; ^5^ Faculty of Mechanical Engineering and Naval Architecture University of Zagreb Zagreb Croatia; ^6^ Department of Chemical Engineering School of Industrial Engineering University of Málaga Málaga 29071 Spain; ^7^ Department of Inorganic Chemistry Faculty of Science Interuniversitary Institute for Research in Biorefineries I3B University of Malaga Málaga 29071 Spain; ^8^ School of Mathematical Sciences Sunway University Bandar Sunway Petaling Jaya Selangor Darul Ehsan 47500 Malaysia; ^9^ Department of Mathematics & Physics North South University Dhaka 1229 Bangladesh; ^10^ Chemical Engineering Department Faculty of Engineering Diponegoro University Semarang Indonesia; ^11^ Institute of Research and Development Duy Tan University Da Nang Vietnam; ^12^ School of Engineering & Technology Duy Tan University Da Nang Vietnam; ^13^ Institute of Engineering HUTECH University Ho Chi Minh City Vietnam; ^14^ PATET Research Group Ho Chi Minh City University of Transport Ho Chi Minh City Vietnam

**Keywords:** biodiesel, dual‐fuel diesel engine, emission reduction, energy efficiency, hydrogen enrichment, sustainable development goal

## Abstract

This study investigates the influence of hydrogen (H_2_) enrichment on the performance, combustion, and emission characteristics of a dual‐fuel diesel engine operated with Mahua biodiesel/diesel blend (BDf20), in which H_2_ is injected into the intake manifold at flow rates of 4, 6, 8, 10, and 12 L min^−1^ under varying engine loads. As a result, the optimum engine performance is achieved at 10 L min^−1^ H_2_. At a peak load of 5.02 kW, BDf20 + H_2_ (10 L min^−1^) improves brake thermal efficiency (BTE) by 16.75%, and reduces brake specific fuel consumption (BSFC) by 10.83% compared to conventional diesel. For emission characteristics, unburnt hydrocarbons (HC), carbon monoxide (CO), and carbon dioxide (CO_2_) decrease by 42.65, 44.74, and 20.91%, respectively, although NOx emissions increased by 17.1% due to higher combustion temperatures. Moreover, combustion characteristics show a 9.91% rise in peak in‐cylinder pressure, a 20.82% increase in heat release rate, and an 8.26% longer ignition delay period. The results confirm the effectiveness of H_2_ enrichment in improving combustion performance while significantly reducing pollutant emissions, showing that combining H_2_ with biodiesel enhances the global Sustainable Development Goals (SDG) by advancing clean and renewable energy solutions.

## Graphical abstract

This study explores hydrogen enrichment in a dual‐fuel diesel engine fuelled with a Mahua biodiesel/diesel blend. At 10 L min^−1^ hydrogen flow, engine efficiency rises while fuel consumption and major emissions decline, though NOx increases. The findings highlight hydrogen's role in cleaner combustion and its contribution to advancing Sustainable Development Goals.

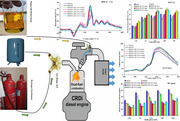

## 1 Introduction

In the current scenario, internal combustion engines (ICEs) are used in a variety of sectors, including energy production as well as transportation. Such combustion engines, especially diesel‐powered ones, have become widespread worldwide due to their high thermal performance, operational stability, and adaptability to a wide range of conditions.^[^ ^1^, ^2^ ^]^ Diesel vehicles are popular in heavy‐duty and commercial applications due to their endurance and ability to generate significant power at low speeds. They are important in industries such as agriculture, transportation, and manufacturing.^[^ ^3^, ^4^ ^]^ However, ICE's dependency on traditional gasoline and diesel fuels reveals remarkable concerns. The continued use of fossil fuels has raised concerns about the depletion of limited oil and gas supplies, pointing to an impending energy catastrophe.^[^ ^5^, ^6^, ^7^ ^]^ In addition, the combustion of fossil fuels emits significant amounts of greenhouse gases (GHGs) and other pollutants such as carbon monoxide (CO), unburned hydrocarbons (HC), carbon dioxide (CO_2_), nitrogen oxides (NO_x_), sulfur oxides (SO_x_), and particulate matter (PM), which harm the ecosystem by causing climate change, degraded air standards, and negative effects on people and the environment.^[^ ^8^, ^9^, ^10^ ^]^ The growing demand for energy and the critical need to reduce greenhouse gas (GHG) and pollutant emissions have prompted researchers, scientists, and other technical experts to investigate renewable and environmentally acceptable alternatives to fossil fuels.^[^ ^11^, ^12^, ^13^ ^]^ Among these possibilities, sustainable biofuels and hydrogen (H_2_) are emerging as the leading contenders, with the potential to minimize dependence on oil and natural gas and eliminate GHGs, contributing to a cleaner and greener future. This transition is in line with global efforts to combat global warming, increase energy security, and achieve Sustainable Development Goals (SDGs), such as clean energy (SDG 7) and climate action (SDG 13).^[^ ^14^, ^15^, ^16^ ^]^

The rapid combustion of H_2_ and carbon‐free molecules increases its attractiveness as an alternative energy source. H_2_ is a clean‐burning fuel that emits only water vapor throughout combustion, thus avoiding dangerous GHG and other hazardous pollutants.^[^ ^17^, ^18^ ^]^ The rapid flame propagation of H_2_ results in shorter ignition times, which improves engine performance and minimizes smoke and PM generation. This faster and more thorough combustion results in more environmentally friendly emissions, which comply with stringent pollution guidelines alongside environmental sustainability goals.^[^ ^19^, ^20^ ^]^ However, the ignition process for producing H_2_ yields higher maximum temperatures, potentially resulting in increased NO_x_ emissions, requiring an advanced emission mitigation technology. H_2_ broad combustible zone and fast igniting speed make it an excellent propellant for diesel/gasoline engines and gas turbines.^[^ ^21^, ^22^ ^]^ Because of its broad combustibility spectrum, ignition is possible over a wider variety of air‐fuel ratios, allowing engines to run efficiently in both lean and rich conditions. The rapid rate of combustion of H_2_ enables faster and more thorough ignition, resulting in shortened combustible duration and lower thermal losses. These characteristics allow the engine to run at higher speeds with improved combustion stability. As a result, H_2_ improves thermal efficiency by increasing energy utilization and boosting power output, rendering it an attractive option for efficient, renewable energy systems.^[^ ^23^, ^24^ ^]^

Recent studies have thoroughly investigated the impact of H_2_ enrichment on the efficiency and GHG emissions of diesel vehicles with different A/F ratios, aiming to determine whether the use of H_2_ as a supplemental fuel affects key engine parameters. The influence of lean or rich mixtures was investigated to evaluate the optimal balance between enhanced performance and the difficulty of regulating emissions during H_2_ enrichment.^[^ ^25^ ^]^ In a study by Karagoz et al.^[^ ^26^ ^]^, a comprehensive investigation was conducted to determine the impacts of 30% H_2_ enrichment on the combustion, efficiency, and emission characteristics of a conventional diesel engine. The research findings revealed a mixed effect of H_2_ enrichment on engine efficiency and GHG emissions. The BTE decreased by 6.3%, indicating that the kinetic energy generated by H_2_ does not fully compensate for the energy loss generated by diesel. The non‐toxic ignition characteristics of H_2_, including its carbon‐neutrality, resulted in a significant 68.4% reduction in CO emissions. However, at full load, NO_x_ emissions increased by 51.3% due to higher cylinder temperatures, resulting from faster H_2_ combustibility. In another case, Karagöz et al.^[^ ^27^ ^]^ examined the implications of H_2_ enrichment on a diesel vehicle employing a hydrogen energy share (HES) ranging from 22 to 53%. The results showed significant enhancements in GHG and fuel parameters with increased H_2_ induction through the intake manifold. A 22% HES reduced CO by 67.3% and smoke opacity by 43.6%, while a 53% HES reduced both CO and smoke opacity by 69 and 58.6%, respectively. In terms of combustion dynamics, the investigation showed a substantial increase in the heat release rate (HRR) of 25.77% at HES of 22% and 110.94% at HES of 53%. Similarly, peak cylinder pressure increased by 7.81% for HES of 22% and 36.2% for HES of 53%. The results obtained highlight the H_2_ enrichment's propensity to improve combustibility while successfully reducing greenhouse gas emissions. Yilmaz et al.^[^ ^28^ ^]^ performed an investigation to determine the influence of H_2_ enrichment on combustion parameters associated with a common rail direct injection diesel engine. The addition of H_2_ resulted in a significant increase in cylinder pressure, varying from 1.1 to 11.21%. The ignition delay period (IDP) increased by 52% and 47.06% for H_2_ flow rates of 20 and 40 L min^−1^, respectively, reflecting a prolonged pre‐combustion state resulting from H_2_’s intense diffusivity. However, the investigation found a decrease in HRR from ≈1.54 to 19.55% compared to direct diesel ignition, indicating a regulated combustion energy release. These findings emphasize the H_2_’s intricate role in changing combustion processes, which promotes pressure build‐up and poses difficulties in regulating heat release and ignition dynamics.

Koten^[^ ^29^ ^]^ investigated the efficiency of an upgraded diesel engine‐powered vehicle enhanced with H_2_ inducement of 0.8 L min^−1^ and found a 3% improvement in BTE. This improvement was mainly due to the H_2_’s faster flaming velocity, which speeds up the burning process, and its ability to generate a homogenous air‐fuel combination, resulting in more complete and effective combustion. The research also found that having an optimal H_2_ content substantially decreased CO, HC, and soot emissions, demonstrating H_2_’s ability to enhance overall engine performance and pollutants. Jabbr et al.^[^ ^30^ ^]^ investigated the relationship between key engine performance attributes, including BTE, BSFC, CO, HC, NOx, and soot, and input variables such as HES, IT, and EGR, using a diesel‐powered engine operating in dual‐fuel mode fueled with H_2_‐enriched gaseous fuel. The research objective was to improve the trade‐offs between efficiency and GHG emissions in dual‐fuel operation. The replacement of hydrocarbon‐based diesel fuel and the carbon‐neutral nature of H2 resulted in a remarkable 60% drop in smoke emissions. Additionally, the inclusion of 15% EGR dilutes the injected charge, reducing the oxygen content and the chamber wall temperature. This method contributed to a significant 88% diminution in NO_x_ emissions, overcoming one of the main problems underlying H_2_‐enriched gaseous fuel. Ayad et al.^[^ ^31^ ^]^ used both experimental and computational techniques to assess the impact of H_2_‐enriched gaseous fuel induction on the efficiency of turbocharged spark‐ignition (SI) engines powered by ethanol‐petrol blends. The investigation revealed that adding H_2_ greatly increased ethanol's combustibility spectrum, allowing the combustion system to run more effectively at lower air‐fuel combinations. This leaner operation also improves combustion stability and minimizes EGT, resulting in lower thermal stresses and increased engine endurance. The wider combustibility spectrum afforded by H_2_ contributed to cleaner and improved ignition, highlighting H_2_’s viability as a supplementary fuel alongside ethanol in the modern SI petrol engine operations. Singh et al.^[^ ^32^ ^]^ investigated the efficiency of a dual‐fuel diesel engine fueled with a bio‐CNG/diesel blend including H_2_ supplementation. The data suggest that the introduction of H_2_ increases power, in addition to exergy efficiency, by improving ignition, reducing fuel consumption, and improving overall engine efficiency. The addition of H_2_ promotes cleaner combustion, reducing pollutants such as CO, HC, and particulate matter. Furthermore, exergy efficiency is enhanced because of improved energy conversion, resulting in less heat loss. This suggests that H_2_ supplementation is a viable technique for a more environmentally friendly and effective engine operation, but additional tuning is required. Saaidia et al.^[^ ^33^ ^]^ analyzed the combustibility and engine efficiency of a diesel engine running on H_2_‐enriched CNG. H_2_ fractions of 20 and 30% by volume were studied, and results showed a substantial increase in combustion, especially when used under premixed conditions. Maximum in‐cylinder pressure rose and combustion stability improved, with test fuel blends below 11%. Performance enhancements comprised an 11.3% rise in energy efficiency and a reduction in fuel consumption to 247 g kWh^−1^. The results show that H_2_‐enriched CNG is a possible replacement for traditional diesel fuel, with lower pollutants and higher efficiency.

Considering its sulfur‐free structure, high oxygen content, and sustainable nature, biodiesel is attracting much interest as a potential fuel substitute for diesel produced from conventional fuels. These characteristics make it a more environmentally friendly choice, resulting in lower GHG emissions and better combustion properties.^[^ ^34^ ^]^ However, its lower heating value and higher kinematic viscosity, compared to fossil fuels, can cause malfunctions such as diminished horsepower, greater BSFC, and reduced engine performance.^[^ ^35^ ^]^ Thiruselvam et al.^[^ ^36^ ^]^ investigated H_2_‐enriched waste plastic oil with 5% n‐butanol in a single cylinder CI engine. Using a 30 and 40% blend with H_2_ at 8 and 10 L min^−1^, they observed BTE gains up to 8.22%, and BSFC reductions to 20.89%. CO, HC, and smoke opacity decreased significantly, though NOx rose to 236 ppm. Peak pressure and HRR improved, with 40WPO + nBut5 + H_2_ (10 L min^−1^) delivering optimal results, supporting waste plastic oil as a sustainable, cost‐effective diesel alternative despite NOx challenges. Krishnamoorthi et al.^[^ ^37^ ^]^ explored Cocos nucifera biodiesel with 30 ppm AL_2_O_3_ nanoparticles and H_2_ enrichment (8 and 10 LPM) in a CI engine. The 40 Cocos nucifera biodiesel + AL_2_O_3_ blend with 10 LPM H_2_ improved BTE by 6.86% and reduced BSFC by 25.98%, CO by 54.13%, HC by 15.95 ppm, and smoke by 14.71% while NOx rose by 604 ppm. Enhanced combustion, higher peak pressure, and increased HRR demonstrated the blend's potential as a sustainable, efficient diesel alternative, particularly in tropical regions with abundant coconut‐derived biofuel resources. Thiruselvam et al.^[^ ^38^ ^]^ evaluated the performance and emission characteristics of a diesel engine fueled with palm biodiesel, H_2_‐enriched palm biodiesel, and palm biodiesel with H_2_ and 30 ppm CeO_2_ nanoparticles compared to diesel fuel. Results showed a significantly reduced BSFC (20.68%) and an increase in BTE (7.93%) for palm biodiesel with H_2_ and 30 ppm CeO_2_ nanoparticles compared to the case of diesel fuel. Emissions of CO, HC, NOx, and smoke opacity decreased substantially, meeting Euro VI limits except for NOx. This study recommends palm biodiesel with H_2_ enrichment and CeO_2_ nanoparticles for enhanced efficiency, cleaner emissions, and vibration characteristics close to diesel operations. The same line was also investigated for the use of H_2_‐enriched palm biodiesel as an alternative fuel for diesel engines to improve performance and reduce emissions.^[^ ^39^ ^]^ H_2_ was introduced at 6 and 8 L min^−1^ with palm biodiesel blends, compared to conventional fuel. Results showed that 30% palm biodiesel + 8 L min^−1^ H_2_ reduced BSFC by 19% and increased BTE by 3.4–6.7%. In addition, emissions of CO, HC, and smoke opacity decreased significantly, while NOx slightly increased. The enhanced flammability and combustion of H_2_ enabled efficient lean operation, offering a viable approach for reducing vehicular air pollution and alternative fuel strategies.

Scientists have investigated the use of H_2_‐enriched gaseous fuel in combination with biodiesel as a solution to the aforementioned issues, taking advantage of H_2_’s high energy density and carbon‐neutral flame characteristics. With the aim of improving combustion efficiency and reducing pollution, the latest study has focused on evaluating the cumulative effects of H_2_ and biodiesel on engine performance and emission levels. According to these studies, H_2_‐enriched biodiesel could improve thermal efficiency, reduce CO and HC, and achieve a better balance between ecological footprint and performance, all of which are drawbacks of biodiesel itself. Therefore, H_2_ enrichment represents a viable way to maximize the use of biodiesel in modern diesel engine‐powered vehicles designed to operate on dual‐fuel. **Table** **1** provides a brief review of the literature to date, highlighting the research on H_2_‐enrichment in diesel engine‐powered vehicles made to operate on dual‐fuel.

**Table 1:** Effects of hydrogen‐enrichment on performance, emissions, and combustion characteristics of a diesel engine operating in dual‐fuel mode.

Authors details	Fuel sample	Experimental observations	Performance	Emission	Combustion	Concluding remarks
Kanth et al.^[^ ^40^ ^]^	Honge biodiesel + H_2_	20% biodiesel induced H_2_ at 10 and 13 L min^−1^.	BTE ↑; BSFC ↓; EGT↑	HC↓; CO↓; NOx↑; CO_2_↑; Smoke↓	CP↑; RoHR↑; CD↓; MRPR↑	H_2_ enrichment in the intake air of diesel engines operating on biodiesel blends can enhance performance and reduce certain emissions, although attention should be given to potential increases in NO_x_ emissions
Kanth et al.^[^ ^41^ ^]^	Rice bran oil + Karanja oil + H_2_	10 and 20% biodiesel blends induced H_2_ at 7 L min^−1^	BTE ↓; BSFC ↓; EGT ↑	HC↓; CO↓; NOx↑; CO_2_↑; Smoke↓	HRR↓; CP↑	H_2_ enrichment in diesel engines operating on biodiesel blends enhances performance and reduces certain emissions. Notably, rice bran biodiesel blends showed more significant improvements than karanja biodiesel blends.
Loganathan et al.^[^ ^42^ ^]^	Cashew nut shell biodiesel + H_2_	A 20% biodiesel blend induced H_2_ at 6 L min^−1^	BTE↑; BSFC↓	HC↓; CO↓; NOx↑; Smoke↓	HRR↑; ICP↑	Incorporating di‐ethyl ether into H_2_‐enriched CNS biodiesel positively influences engine performance by enhancing efficiency and reducing certain emissions.
Gad et al.^[^ ^43^ ^]^	Cotton seed biodiesel + kerosene + HHO gas	20% biodiesel + 5 and 10% kerosene additive with 0.3 L min^−1^ induced HHO	BTE↓; BSFC↑; EGT↑	HC↓; CO↓; NOx↑; CO_2_↑; Smoke↓	–	Enriching the intake air with HHO gas in diesel engines fueled by biodiesel blends with kerosene additive enhances performance by increasing thermal efficiency and reducing specific fuel consumption. While beneficial reductions in CO and HC emissions are achieved
Rajak et al.^[^ ^44^ ^]^	H_2_ + diesel+ n‐butanol + di‐ethyl ether	5% H_2_ share induced with a 95% fraction of di‐ethyl ether and n‐butanol	BTE↑; BSFC↑	HC↓; CO↓; NOx↑; CO_2_↑; Smoke↓	HRR↑; ICP↑; IDP↓	Integrating H_2_ into diesel engines operating in dual‐fuel mode offers potential benefits in performance and emissions.
Vasanthakumar et al.^[^ ^45^ ^]^	Ethanol+ diesel+ H_2_	9 L min^−1^ H_2_ induced with 5,10, balance and 15% ethanol‐diesel blend	BTE↑; BSFC↓; BSEC↓	HC↓; CO↓; NOx↑; CO_2_↓; Smoke↓	HRR↑; ICP↑	Enriching the intake air with H_2_ positively influences the performance and emissions of a diesel engine fueled with ethanol‐diesel blends, offering potential benefits for cleaner and more efficient engine operation.
Ranjit et al.^[^ ^46^ ^]^	Jatropha biodiesel + H_2_	Injection pressure variation (175, 205, 235, and 265) bar	BTE ↑; BSFC ↓	HC↓; CO↓; NOx↑; Smoke↓	ICP↑; IDP↓	H_2_ supplementation, combined with optimized injection parameters, can enhance engine performance and reduce certain emissions.
Tarabet et al.^[^ ^47^ ^]^	Eucalyuptus biodiesel + H_2_ + natural gas	100% biodiesel + 85NG–15H_2_; 75NG–25H_2_; 70NG–30H_2_	BTE↓; BSFC↑; EGT↓	HC↓; CO↓; NOx↑; Smoke↓	HRR↓; ICP↓	Supplementing natural gas with H_2_ in a dual‐fuel DI diesel engine with a biodiesel pilot fuel enhances engine performance and reduces emissions. This approach offers a promising pathway toward more sustainable and efficient engine operation.
Chaichan^[^ ^48^ ^]^	Biodiesel + diesel + H_2_	100% biodiesel with 20% H_2_ vol. fraction	BTE↓	HC↓; CO↓; NOx↑; Smoke↓	–	Combining H_2_ supplementation with massive EGR in CIEs offers potential benefits in performance and emission control.
Tosun et al.^[^ ^49^ ^]^	Soyabean biodiesel + H_2_ + Al_2_O_3_	20 and 100% biodiesel with H_2_ induction at 5 L min^−1^	BTE↑; BSFC↓	HC↓; CO↓; NOx↑; CO_2_↑; Smoke↓	HRR↑; ICP↑	Supplementing diesel‐soybean biodiesel blends with H_2_ and nanoparticles enhances engine performance and reduces emissions. This approach offers a promising pathway toward more sustainable and efficient engine operation.
Kanth et al.^[^ ^50^ ^]^	Rice bran oil + H_2_	10 and 20% biodiesel blends with induced H_2_ at 7 L min^−1^.	BTE↓; BSFC↓; EGT↑	HC↓; CO↓; NOx↑; CO_2_↑; Smoke↓	HRR↓; ICP↑	H_2_ enrichment improves the performance of diesel engines running on biodiesel blends while reducing emissions. Among the tested fuels, rice bran biodiesel blends exhibited greater efficiency gains and emission reductions.
Tayari et al.^[^ ^51^ ^]^	Diesel + microalgae Chlorella vulgaris + H_2_	20% microalgae Chlorella vulgaris with 10 L min^−1^ induced H_2_ gas.	BTE↓; BSFC↓	HC↓; CO↓; NOx↑; CO_2_↑; Smoke↓	–	H_2_ supplementation in CVME fuel blends enhances engine performance and reduces emissions, offering a promising alternative to conventional diesel fuels.
Chakraborty et al.^[^ ^52^ ^]^	Diesel + H_2_	100% diesel with 5, 10 L min^−1^ induced H_2_ gas	BTE↑	HC↓; CO↓; NOx↑; CO_2_↓; Smoke↓	HRR↓; ICP↑; IDP↑	H_2_ enrichment in diesel fuel enhances performance and reduces most emissions, while LPG enrichment offers lower NOx emissions, providing a comparative analysis for optimizing engine operations.
Rocha et al.^[^ ^53^ ^]^	Diesel + biodiesel + H_2_	7% biodiesel‐diesel with 5, 15, and 20 L min^−1^ of induced H_2_ gas	VE↓; BSFC↓; EGT↑	HC↓; CO↓; NOx↑; CO_2_↓; Smoke↓	ICP↑; RoHR↑; SOC↓	The study demonstrates that optimizing the H_2_‐biodiesel blend ratio can help strike a balance between performance improvements and emissions reductions. Additionally, the results suggest that H_2_ can be a viable alternative to improve the environmental footprint and operational efficiency of CI engines running on renewable fuels like biodiesel.

Based on the above analysis, the integration of H_2_ with biodiesel in a dual‐fuel strategy emerges as a viable solution to address the inherent limitations of biodiesel in CI engines. Owing to its high heating value, wide flammability range, rapid flame speed, and carbon‐free molecular structure, H_2_‐supplementation enhances ignition quality. It compensates for the lower calorific value and higher viscosity of biodiesel. This enrichment improves combustion efficiency, increases brake thermal efficiency (BTE), and reduces key exhaust pollutants, including CO, HC, and smoke opacity. The present study extends beyond earlier biodiesel‐H_2_ enrichment work by systematically evaluating the combined influence of H_2_ induction and biodiesel blending under multiple engine loads, with a detailed assessment of performance, combustion, and emission parameters. The results provided a technical basis for optimizing dual‐fuel operation to meet sustainable development goals, particularly in the context of cleaner energy and reduced vehicular emissions.

## 2 Experimental Section

### 2.1 Source of Mahua Oil

Mahua oil was extracted from the seeds of the Madhuca indica tree, also referred to as Mahua. This drought‐adapted, non‐edible oilseed‐bearing plant grown primarily in tropical and subtropical parts of India, Nepal, Bangladesh, and Sri Lanka. Mahua trees produced small, yellowish‐green fruits containing oil‐rich seeds with oil concentrations ranging from 30 to 40% by weight.^[^ ^54^ ^]^ In the present investigation, Mahua oil was procured from Expo Essential Oils in Mayur Vihar Phase 1, New Delhi. The base oil was then purified and processed to eliminate contaminants, including free fatty acids (FFA), phospholipids, and moisture for biodiesel synthesis. The FFA composition of Mahua oil methyl ester is depicted in **Table** **2**.

**Table 2:** Fatty acid composition analysis of Mahua oil.

Fatty acid component	Fatty acid structure	Structure formula	FFA wt. [%]
Oleic acid	C18:1	C_18_H_34_O_2_	37.3
Palmitic acid	C16:2	C_16_H_32_O_2_	24.5
Stearic acid	C18:0	C_18_H_36_O_2_	22.4
Linoleic acid	C18:2	C_18_H_34_O_2_	14.4
Arachidic acid	C20:0	C_20_H_40_O_2_	1.4

### 2.2 Preparation of Mahua Biodiesel

The transesterification process converted crude Mahua oil into biodiesel by combining it with methanol using a catalyst. The procedure started with the separation of the oil from the Mahua seeds by mechanical pressing. The crude oil undergrown degumming and filtering to eliminate contaminants such as phospholipids, FFA, and moisture, all of which could reduce the efficiency of transesterification. The transesterification reaction used NaOH or H_2_SO_4_, relying upon the FFA content of the Mahua oil. As the FFA content was high, an acid esterification pre‐treatment phase was conducted prior to the base‐catalyzed transesterification to avoid soap production.^[^ ^55^ ^]^ The chemical process occured by mixing Mahua oil and methanol in a molar ratio of about 6:1, introducing 0.5–1% (w/w) of catalyst at a controlled temperature (50–60 °C), and stirring for about 2 h. After completion, the blend get to settle, resulting in the separation of two phases, including the topmost phase of methyl esters (biodiesel) and glycerol. The resulting biodiesel was then refined by washing it with warm water to remove the remaining catalyst alongside unprocessed methanol, and subsequently air‐dried to remove moisture. To ensure compliance with fuel quality norms, important physicochemical parameters, including kinematic viscosity, specific gravity, heating value, cetane index, flash point, fire point, and pour point of produced biodiesel, were tested.

### 2.3 Physio‐Chemical Characteristics of Test Fuels

The study of the physicochemical parameters of Mahua biodiesel‐diesel fuel blends alongside H_2_‐enriched gaseous fuel was essential to determine their suitability in diesel vehicles. Produced Mahua biodiesel was blended with standard diesel fuel to improve combustion characteristics and compliance with currently available diesel engine‐powered vehicles. The blending procedure included a combination of biodiesel with diesel fuel in specific proportions, including B20 (20% Mahua biodiesel + 80% diesel), depending on the intended use and engine compatibility. A higher cetane index increased ignition quality, resulting in better combustibility, while higher flash and fire points increased fuel safety. However, the cold flow properties of biodiesel, such as higher cloud and pour points, presented issues in low‐temperature environments, requiring additives or optimized mixing. **Table** **3** depicts the physio‐chemical characteristics of diesel fuel, Mahua biodiesel (Bf100), and Mahua biodiesel/diesel blend (BDf20) as per ASTMD‐6751 standard.

**Table 3:** Physio‐chemical characteristics of diesel fuel, BD_f_100, and BDf20 as per ASTMD‐6751 standard.

Property	Unit	Diesel	BD_f_100	BDf20	ASTM D6751
SG@15 °C	kg m^−3^	842.0	898.0	864.0	850–900
KV@40 °C	CSt	2.4	3.37	2.88	1.9–6.0
FLP	°C	63.0	111	89.0	≤130.0
FIP	°C	71.0	132	97.0	≤200
CV	MJ/kg	41.6	38.2	39.4	≥35.0
CI	–	48	54.0	51.0	≥50.0
PP	°C	−6.0	4.0	−1.0	−15 to 10

**SG‐ specific gravity; KV‐ kinematic viscosity; FLP‐ flash point; FIP‐ fire point; CV‐ calorific value; CI‐ cetane index; PP‐ pour point.

For this study, H_2_ gas was obtained from Sri Venkateswara Carbonic Gases Private Limited in Athipalayam, Coimbatore, India. H_2_ was a carbon‐neutral fuel that removed CO_2_, CO, and particulate matter. However, its elevated adiabatic flame temperatures (2046 °C) could boost NO_x_ formation. Hydrogen's lower heating value (120 MJ kg^−1^) contributed significantly to energy release, although its modest volumetric energy density at 15 °C and 100 kPa of about 10.7 MJ m^−3^ required high‐pressure transport. **Table** **4** briefly elaborates on the properties of H_2_ gaseous fuel.

**Table 4:** Properties of hydrogen (H_2_) gaseous fuel.

Properties	Units	Values
Density at 15 °C and 100 kPa	kg m^−3^	0.089
Adiabatic flame temperature	°C	2046.0
Lower heating value	MJ kg^−1^	120.0
Carbon content	Mass%	0.0
Auto ignition temperature	°C	586.0
Octane number	–	130.0
Flame velocity	cm s^−1^	270.0
Flammability limits (vol. in the air)	%	4–74
Energy density at 15 °C and 100 kPa	MJ m^−3^	10.7
Diffusivity in air	cm^2^ s^−1^	0.65

## 3 Experimental Setup

### 3.1 Engine Setup

The current investigation used a water‐cooled, four‐stroke, single‐cylinder CRDi diesel engine (Kirloskar make TV1_model) with a rated speed of 1500 rpm and a maximum power output of 5.02 kW. Detailed engine specifications are depicted in **Table** **5**. This engine configuration was chosen to test the combustibility, efficiency, as well as greenhouse gas emissions of different combinations of fuels under standardized testing conditions.

**Table 5:** Test engine technical specifications and details.

Engine parameters	Engine specification details
Make and model	Kirloskar Make TV1_Model
Number of cylinders	1‐Cylinder; 4‐Stroke
Stroke (mm) × Bore (mm)	(110 × 87.50) mm
Cooling system	Water cooled
Swept volume (cc)	661.45 cc
Injection timing (°bTDC)	24 °bTDC
Injector opening pressure (bar)	600 bar
Rated power and speed	5.02kW@1500 rpm
Compression ratio	18.0:1
Dynamometer used	Eddy‐current type

**Figure 1:**
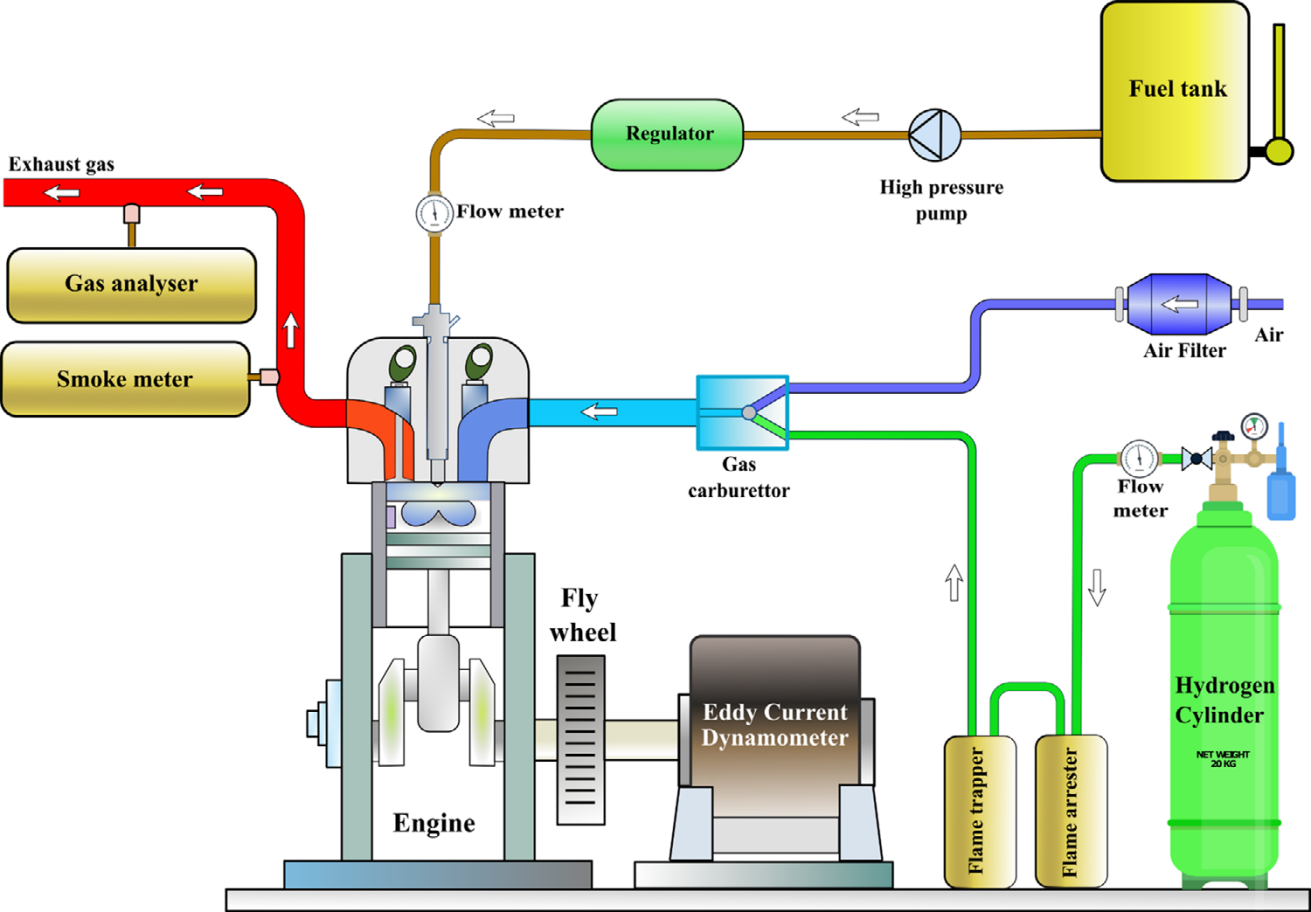
Graphical representation of the experimental test rig.

**Figure** **1** shows the experimental configuration in animated form. The tested CRDi engine consisted of a high‐pressure fuel injection system, a pressure regulating valve, a rail piping enabling fuel dispersion, and a rail pressure monitoring system. The pressure transducer interfaced with the Electronic Control Unit to monitor and controlled the diesel rail pressure to ensure maximum engine performance. The load was applied using a water‐cooled eddy current dynamometer, allowing precise control of the engine workload. A crank angle encoder was positioned on the crankshaft to accurately determine its orientation and rotational velocity for analyzing the engine's efficiency. Furthermore, a piezoelectric pressure transducer was positioned onto the cylinder head to record actual pressure variations inside the cylinder, aiming to determine combustion parameters, ignition delay period, and heat release rate. The data acquisition system received pressure signals from a piezoelectric transducer mounted onto the piston head that continually captured in‐cylinder pressure fluctuations throughout the combustion process. This information then calculated key combustion parameters such as heat release rate (HRR), peak cylinder pressure, and ignition delay period.

K‐type thermocouples were used to determine the temperature of different engine parts because of their excellent precision and large temperature operating range. These thermocouples had been carefully placed to enable actual temperature observation and effective engine performance analysis. A thermocouple sensor mounted on the exhaust manifold near the cylinder head measured the exhaust gas temperature (EGT). This positioning allowed accurate measurement of temperature fluctuations in the pollutants, analyzing combustion efficacy, and measuring heat dissipation across the entire system. An AVL five‐gas detector had been utilized for continuous monitoring alongside characterized diesel‐powered vehicle exhaust greenhouse gases, enabling accurate detection of critical contaminants including CO, HC, and NO_x_. The instrument used modern electronic sensors to provide accurate real‐time data for greenhouse gas assessment. To ensure repeatability and reliability of the experimental results, each test condition was performed three times under identical operating parameters. The reported values for performance, emissions, and combustion characteristics represented the arithmetic mean of these repeated measurements.

As shown in Figure 1, the H_2_ pipeline had a number of vital parts that facilitated the precise and secure transportation of H_2_ into the cylinder. A pressure regulator was fitted to regulate and maintain the H_2_ pressure between the storage tank and the combustion chamber inlet unit. The flowmeter accurately detected and continuously tracked the H_2_ flow rate, allowing precise regulation of the volume of H_2_ delivered within the ignition chamber. To improve operational safety, a flow control valve had been employed for controlling and modifying the H_2_ induction based on scientific parameters. To protect against backfire, the supply pipeline also included a flame arrester and a flame trap. The flame arrester removed heat from any possible flame front, preventing combustion from travelling back towards the H_2_ supply pipeline. Similarly, the flame trap protected against fire flashback, thus maintaining the stability of the H_2_ storage and distribution systems. The pressurized tank was designed to store H_2_ at an elevated pressure of 300 bar, providing an appropriate supply over the engine test‐rig setup. A pressure regulator had been employed to lower the high pressure to about 5 bar, allowing a controlled as well as consistent supply of H_2_ through the engine inlet manifold.

### 3.2 Hydrogen Energy Contribution

The Hydrogen Energy Contribution (HEC) estimated the amount of energy produced by H_2_ in relation to conventional fuels like diesel and biofuels. This parameter was crucial for evaluating H_2_’s contribution to lowering GHG and increasing energy efficiency in power generation. It was influenced by the H_2_ energy content and system performance, including the hybrid energy generating system's distinctive layout.^[^ ^56^ ^]^ The HEC could be assessed by examining the system's energy balance. The H_2_ energy contribution at different engine loads was calculated theoretically using experimental measurements of H_2_ flow rate and diesel/biodiesel fuel consumption. The mass flow rate of H_2_ was measured using a calibrated flow meter. The energy input from H_2_ was then determined by multiplying the H_2_ mass flow rate by its lower heating value (LHV). Similarly, the energy input from the diesel/biodiesel blend was calculated using the fuel consumption rate and its corresponding LHV. The H_2_ energy contribution percentage was obtained by dividing the H_2_ energy input by the total fuel energy input (sum of H_2_ and diesel/biodiesel blend energies) and multiplying by 100. In general, Equation (1) was used for estimating the H_2_’s energy contribution to the overall power generated by the system, which was presented below:

(1) $$HEC\left(\%\right)=\frac{E_{H}}{E_{\mathrm{Total}}}\times 100$$

**Figure 2:**
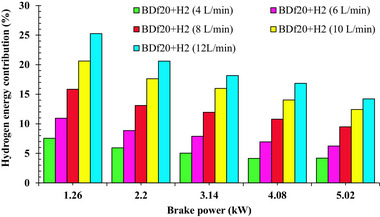
Fluctuation in the HEC for multiple test fuel samples BDf20 + H_2_ (4 L min^−1^), BDf20 + H_2_ (6 L min^−1^), BDf20 + H_2_ (8 L min^−1^), BDf20 + H_2_ (10 L min^−1^), and BDf20 + H_2_ (12 L min^−1^) at different brake power (kW).

Here: *E*_H_ represents the energy output from H_2_, *E*_H =_ ${\dot{m}}_{H2}LVH_{H2}$; ${\dot{m}}_{H2}$ is the mass flow rate of H_2_ in kg h^−1^ (measure experimentally via flow meter), and *LVH*_*H*2_ is a lower heating value of H_2_ in MJ kg^−1^. Similarly, *E*_Total_ represents total energy output, which includes both H_2_ and the diesel/biodiesel fuel, as given in Equation (2).

(2) $$E_{\mathrm{Total}=}\;{\dot{m}}_{H2}LVH_{H2}+{\dot{m}}_{fuel}LVH_{fuel}$$

where, ${\dot{m}}_{fuel}$ is the mass flow rate of diesel/biodiesel fuel in kg h^−1^ (measured experimentally via fuel consumption rate) and *LVH_fuel_* was lower heating value of diesel/biodiesel blend in MJ kg^−1^ respectively. **Figure** **2** depictes the fluctuation in the HEC for multiple test fuel samples, BDf20 + H_2_ (4 L min^−1^), BDf20 + H_2_ (6 L min^−1^), BDf20 + H_2_ (8 L min^−1^), BDf20 + H_2_ (10 L min^−1^), and BDf20+H_2_ (12 L mi^−1^n) at various workloads of 1.26 , 2.2 , 3.14 , 4.08 , and 5.02 kW.

### 3.3 Uncertainty Analysis

Quantification errors had been an inevitable part of any scientific study and were affected by a variety of components, such as the sensitivity and calibration of the measuring equipment, ambient conditions, and the accuracy of the results obtained during the course of the investigation. These errors added to uncertainty, resulting in an important measure of the precision and accuracy of experimental results. Uncertainty could develop in scientific studies as a result of either random or systematic errors. The standard approach represented uncertainty as a percentage uncertainty, offering an approximate indication of the error in the observed variable. Specific uncertainties in parameters were determined using the Kline and McClintock uncertainty propagation method,^[^ ^57^ ^]^ which evaluated error propagation using algebraic interpretations. Once the uncertainties associated with each observation were known, the overall uncertainty concerning the test setup was obtained by applying a suitable error propagation equation. Throughout the present investigation, the percentage uncertainties associated with several variables were established to determine experimental accuracy. Uncertainty was critical for evaluating the results of experiments, enhancing the precision of data, and refining methods for testing. Considering the sensitivity of each input variable to the output, the overall uncertainty is expressed in Equation (3):

(3) $$U_{R}=\sqrt{\sum_{i=1}^{n}{\left(S_{\mathrm{Xi}}\times U_{\mathrm{Xi}}\right)}^{2}}$$

Here, U_R_ represented the total uncertainty in the result “R”;*U_Xi_* was the uncertainty in the independent variable *Xi*; *S_Xi_* was the sensitivity coefficient of “R” with respect to *Xi*.

Considering parameters derived from multiple measurements, the root‐mean‐square method was applied as reported in earlier studies:^[^ ^58^ ^]^

(4) $$U_{\mathrm{total}}\;\sqrt{U_{1}^{2}+U_{2}^{2}+U_{3}^{2}+U_{4}^{2}+\cdot \cdot \cdot \cdot +U_{n}^{2}}$$

In Equation (4), U_1_, U_2_,…, U_n_ were the uncertainties related to various observed characteristics, such as specific fuel consumption, greenhouse gases, engine output indicators, and fluctuations in temperature. Numerous investigations had been conducted to examine the uncertainty linked to key performance and pollutant measurements, such as brake thermal efficiency (BTE), brake specific energy consumption (BSEC), carbon monoxide (CO), carbon dioxide (CO_2_), hydrocarbons (HC), smoke opacity, and oxide of nitrogen (NO_x_). The total uncertainty (U_total_ or Δ) had been calculated using a defined error propagation procedure to ensure reliable and precise information analysis.

(5) $$\begin{array}{c} \Delta =\sqrt{BTE^{2}+BSEC^{2}+CO^{2}+C{O_{2}}^{2}+HC^{2}+Smokeopacity^{2}+N{O_{x}}^{2}} \end{array}$$

(6) $$\Delta =\sqrt{0.64^{2}+1.41^{2}+1.0^{2}+0.2^{2}+0.6^{2}+0.9^{2}+1.0^{2}}=\pm 2.3$$

**Table** **6** displays the measured parameters, instruments used, range, least count, and uncertainty of different measurement apparatus.

**Table 6:** Assessment of uncertainty over a wide range of testing equipment.

Measured parameters	Instrument used	Range	Least count	Uncertainty [%]
Load (N)	Load cell	0–1000 N	±0.1 N	±0.2
Fuel flow rate (kg sec^−1^)	Digital weighing balance	0–1 kg	±0.01 kg	±0.5
Air flow rate (kg sec^−1^)	U‐tube manometer	0–100 mm	±1.0 mm	±1.0
Brake power (kW)	Torque sensor & tachometer	0–10 kW	±0.01 kW	±0.3
Exhaust gas temperature (°C)	K‐type thermocouple	0–1200 °C	±1.0 °C	±0.75
Cylinder Pressure (bar)	Piezoelectric pressure sensor	0–100 bar	±0.1 bar	±0.5
NO_x_ (ppm)	AVL 444 di‐gas analyser	0–5000 ppm	±10.0 ppm	±1.0
CO (%)	AVL 444 di‐gas analyser	0–10%	±0.01%	±1.2
UBHC (ppm)	AVL 444 di‐gas analyser	0–10 000 ppm	±5.0 ppm	±1.4
Smoke Opacity (%)	AVL 437‐C smoke meter	0–100%	±1.0%	±1.0

## 4 Results and Discussion

### 4.1 Performance Characteristics

#### 4.1.1 Brake Thermal Efficiency

**Figure 3:**
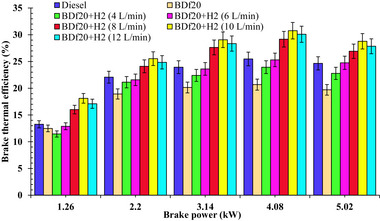
BTE (%) with respect to engine brake power (kW) for diesel, BDf20 and BDf20 + H_2_ gas flow rates at (4, 6, 8, 10, and 12 L min^−1^).

Brake thermal efficiency (BTE) is an important performance metric that measures an engine's capacity to convert the chemical energy of conventional diesel into usable mechanical energy. This is modulated by fuel composition, combustible properties, and engine operational conditions. **Figure** **3** shows the BTE across all test fuels of diesel fuel, BDf20, BDf20 + H_2_ (4 L min^−1^), BDf20 + H_2_ (6 L min^−1^), BDf20 + H_2_ (8 L min^−1^), BDf20 + H_2_ (10 L min^−1^), and BDf20 + H_2_ (12 L min^−1^) at different engine workloads of 1.26, 2.2, 3.14, 4.08, and 5.02 kW.

For each blender studied, the BTE shows an upward trend as the engine workload increases, culminating in maximum load conditions. This observed trend is explained by the increased combustion efficiency at higher workloads, where the fuel‐air mixture, along with ignition, is facilitated by greater turbulence and higher in‐cylinder temperature. The BTE for BDf20 at load conditions of 1.26, 2.20, 3.14, 4.08, and 5.02 kW was recorded as 12.49, 18.93, 20.14, 20.68, and 19.70%, respectively. These values were lower by 5.8, 14.21, 15.9, 18.83, and 20.06% compared to diesel fuel, which exhibited BTEs of 13.26, 22.07, 23.94, 25.48, and 24.65% under the same load conditions. The main causes of this decrease are the increase in kinematic viscosity, specific gravity, and reduction in calorie content of biodiesel, which negatively affect fuel vaporization and spray formation.^[^ ^59^ ^]^ Partial ignition occurs due to an inadequate fuel‐air mixing caused by poor atomization, reducing the energy conversion rate. Additionally, BDf20, with a higher oxygen concentration, produces less thermal energy during ignition, reducing thermal efficiency.

Following H_2_ enrichment, especially at 8 and 10 L min^−1^ input, the BTE shows an increasing pattern. The BTE of BDf20 + H_2_ (8 L min^−1^) and BDf20 + H_2_ (10 L min^−1^) was found to be 9.17 and 16.75% higher, respectively, compared to conventional fuel at a maximum load of 5.02 kW. The significantly lower heating value (LHV) of H_2_, which provides an improved energy content per unit mass, and its faster ignition rate are responsible for this performance improvement. The higher flame propagation velocity of H_2_ allows for faster and more thorough ignition, thereby boosting energy utilization and thermal performance.^[^ ^60^ ^]^ Conversely, a small decrease in BTE was observed for BDf20 + H_2_ (12 L min^−1^), which was found to be 5.57, 2.66, 2.43, 2.15, and 3.26% lower than that for BDf20 + H_2_ (10 L min^−1^) for all loads at 1.26, 2.2, 3.14, 4.08, and 5.02 kW, respectively. The reduction in BTE at H_2_ (12 L min^−1^) compared to H_2_ (10 L min^−1^) could be attributed to the displacement of intake air by excess H_2_, which lowers the availability of oxygen for complete combustion, thereby increasing mixture inhomogeneity and creating over‐lean or over‐rich zones. These conditions, coupled with altered combustion phasing and higher heat losses to the walls, reduce effective heat release and overall energy conversion efficiency. In addition, the reduction may be due to the increased reactivity and efficient ignition of H_2_, which utilizes a substantial amount of the available oxygen in the combustion zone. Higher in‐cylinder temperatures, together with fluctuations in the air/fuel equivalence ratio, could result in localized rich zones and greater heat loss. Such circumstances could prevent complete fuel combustion, which results in partial oxidation and a slight reduction in thermal efficiency.^[^ ^61^ ^]^ The air/fuel equivalence ratio could also be altered by extreme H_2_ enrichment, resulting in localized rich zones and higher thermal deformations, further reducing BTE. Since H_2_ has a higher energy density and a better power‐to‐heat generation ratio than conventional fuels, it significantly improves ignition performance when added as a supplementary fuel to diesel engines.

By promoting improved flame dispersion and reducing localized fuel‐rich regions, this uniform dispersion minimizes partial combustion and improves combustion performance.^[^ ^62^ ^]^ A shorter ignition delay period (IDP) and better HRR result from this faster ignition, which increases BTE. The improved combustion properties, ranging from lower cycle‐to‐cycle variability to a higher energy conversion rate compared to conventional diesel, are the reasons for the higher BTE observed in H_2_‐enriched dual‐fuel engines. At maximum load of 5.02 kW, the BTE for BDf20, BDf20 + H_2_ (4 L min^−1^), BDf20 + H_2_ (6 L min^−1^), BDf20 + H_2_ (8 L min^−1^), BDf20 + H_2_ (10 L min^−1^), and BDf20 + H_2_ (12 L min^−1^) was 19.7, 22.78, 24.76, 26.91, 28.78 and 27.85%, respectively, resulting in 20.06, 7.59%↓ and 0.45, 9.17, 16.75, and 12.98%↑ higher than that of diesel fuel, with a BTE of 24.65%. Similar findings were observed by Yilmaz et al.^[^ ^63^ ^]^ who found that H_2_ enrichment improved combustion properties equivalent to BTE. The results support the claim that the considerable caloric content and fast flame dispersion velocity of H_2_ improve energy utilization, improving engine performance.

#### 4.1.2 Brake‐Specific Energy Consumption

**Figure 4:**
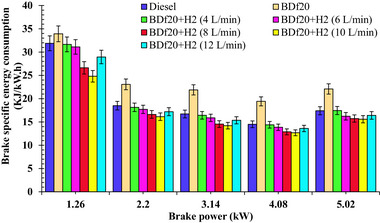
BSEC (kJ kWh^−1^) with respect to engine brake power (kW) for diesel, BDf20 and BDf20 + H_2_ gas flow rate at (4, 6, 8, 10, and 12 L min^−1^).

The energy needed to produce a single unit of braking power output in a diesel engine is known as brake‐specific energy consumption (BSEC). It is a key metric, along with performance, for assessing the cost‐effectiveness of a fuel. Higher thermal performance and improved fuel economy are indicated by lower BSEC values, as more of the energy supplied is converted into productive power. Several variables, such as engine workload, ignition parameters, fuel quality, and operating conditions, affect BSEC. Due to the rapid ignition velocity, greater dispersion and higher combustion effectiveness, H_2_ upgrading has been shown to minimize BSEC, which reduces energy loss and improves engine performance.^[^ ^59^ ^]^ **Figure** **4** shows the BSEC for all test fuels as diesel fuel, BDf20, BDf20 + H_2_ (4 L min^−1^), BDf20 + H_2_ (6 L min^−1^), BDf20 + H_2_ (8 L min^−1^), BDf20 + H_2_ (10 L min^−1^), and BDf20 + H_2_ (12 L min^−1^) at different engine loads of 1.26, 2.2, 3.14, 4.08, and 5.02 kW, respectively.

As engine load increases, BSEC decreases because greater workloads enhance energy consumption alongside combustion effectiveness. Higher BSEC values are due to partial combustion plus thermal losses at lower workloads. Improved air‐fuel mixture, higher in‐cylinder temperature, and enhanced combustion distribution also increase energy consumption as workload rises, lowering BSEC.^[^ ^60^ ^]^ The most effective threshold for achieving the best combustionity and performance attributes among different fuel formulations could be 90%, which is the best possible load level where BSEC achieves the lowest value among all examined test fuel blends. BSEC for BDf20 was found to be 33.92, 23.07, 21.89, 19.42, and 22.08 KJ kWh^−1^, which were found to be 6.37, 24.77, 31.00, 34.02, and 26.38% higher than that of pure diesel fuel at 1.26, 2.2, 3.14, 4.08, and 5.02 kW, respectively. The lower LHV of BDf20 requires additional fuel to produce an equivalent amount of power, which represents the main cause behind the elevated BSEC. Furthermore, the increased viscosity and density of biodiesel result in inadequate fuel vaporization, which causes slow burning than ideal air‐fuel mixture.^[^ ^61^ ^]^ All of these variables work together to increase BSEC, which lowers BDf20's total fuel effectiveness as opposed to conventional fuel.

Because of the low LHVs of BDf20, more BDf20 is needed to provide the same amount of thermal energy as diesel fuel. This is compounded by the poor vaporization properties of biodiesel. To facilitate fuel vaporization during the combustion process, heat is extracted from the cylinder walls, which cools the combustion chamber. Oil droplets cannot fully evaporate upon contact with these lower temperature zones due to the cooled environment. In contrast to conventional diesel, this prematurely vaporized fuel results in partial ignition, which reduces overall combustion performance and contributes to increased energy consumption and reduced thermal efficiency.^[^ ^62^ ^]^ However, the BSEC of BDf20 is improved with H_2_ induction; as the H_2_ concentration increased, the BSEC decreased. Compared with diesel fuel at loads of 1.26, 2.2, 3.14, 4.08, and 5.02 kW, the BSEC of BDf20 + H_2_ (8 L min^−1^) is 16.50, 10.16, 12.97, 11.18, and 9.95%, respectively, while that of BDf20 + H_2_ (12 L min^−1^) is found to be 9.22, 6.98, 8.14, 6.14, and 6.13%, respectively, compared to diesel fuel. Due to its higher calorific content and quicker rate of ignition, H_2_ reduces BSEC when added to BDf20. H_2_ can burn more quickly and efficiently because of its higher flame velocity, and it can also burn steadily under a wider range of working conditions due to its extended combustibility range. Additionally, the shorter extinction interval of H_2_ promotes more thorough burning, which lowers energy waste and boosts thermal performance, resulting in a lower BSEC than in cases without H_2_ support.^[^ ^60^ ^]^

It can be seen in Figure 4 that the combustibility properties of the BDf20 blend improve with H_2_ enrichment, especially at a 10 L min^−1^ flow rate. Subsequently, at an H_2_ induction at 12 L m^−1^, the BSEC begins to increase because of a substantial decrease in oxygen levels within the combustion zone. The excess H_2_ removes the oxygen readily available for BDf20 ignition, resulting in partial combustion and reduced thermal performance. This effect is exacerbated by the higher HRR, resulting in local overheating and greater heat losses, counteracting the advantages of H_2_ enrichment.^[^ ^42^ ^]^ Moreover, the increase in BSEC for H_2_ (12 L min^−1^) compared to H_2_ (10 L min^−1^) arises from the same mechanism as observed in BTE trends, i.e., excessive H_2_ displaces intake air, thereby reducing oxygen availability, while mixture non‐uniformity leads to incomplete combustion. Combined with altered combustion and increased heat losses, these effects reduce effective energy conversion, offsetting the benefits achieved at the optimal enrichment level of 10 L min^−1^. At maximum load of 5.02 kW, BSEC for BDf20, BDf20 + H_2_ (4 L min^−1^), BDf20 + H_2_ (6 L min^−1^), BDf20 + H_2_ (8 L min^−1^), BDf20 + H_2_ (10 L min^−1^), and BDf20 + H_2_ (12 L min^−1^) was 22.08, 17.45, 16.22, 15.73, 15.58, and 16.4 KJ kWh^−1^, respectively, which were 26.38%↑, 0.11%↓, 7.15%↓, 9.95%↓, 10.83%↓ and 6.13%↓ than diesel fuel (BSEC of 17.47 KJ kWh^−1^), respectively. These results are similar to those obtained by Juknelevicius et al.^[^ ^64^ ^]^

#### 4.1.3 Exhaust Gas Temperature

**Figure 5:**
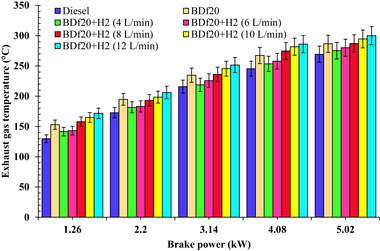
EGT with respect to engine brake power (kW) for diesel, BDf20 and BDf20 + H_2_ gas flow rate at (4, 6, 8, 10, and 12 L min^−1^).

Exhaust Gas Temperature (EGT) is an important parameter that provides useful information on the thermal conditions during ignition, making it essential for identifying pollutant formation, especially NO_x_. Increasing flame temperatures often results in increased NOx production owing to the increased thermal energy offered by the chemical reaction between nitrogen and oxygen. Thus, EGT analysis is a key component for analyzing engine effectiveness as well as the exhaust pollutant performance in various test‐fuel engine configurations.^[^ ^42^ ^]^ **Figure** **5** depicts the effect of multiple fuel samples, such as diesel fuel, BDf20, BDf20 + H_2_ (4, 6, 8, 10, and 12 L min^−1^), on EGT under different engine operating conditions of 1.26, 2.2, 3.14, 4.08, and 5.02 kW, respectively.

From Figure 5, it can be seen that the EGT increases as the workload increases due to the faster‐injected fuel, which generates more heat throughout the combustion chamber. When the amount of fuel supplied increases, so does combustion, resulting in a higher temperature. Furthermore, the EGT of the blended test‐fuel BDf20 was found to be marginally superior relative to that of conventional fuel, because of the oxygen content of BDf20, which promotes improved ignition and therefore increases the in‐cylinder wall temperature. The increased kinematic viscosity, along with inadequate atomization of biodiesel, causes the development of larger fuel droplets, resulting in partial ignition throughout the premixed stage of ignition. As a result, a considerable amount of fuel stands idle and continues burning during the diffusion stage of ignition. The prolonged process of combustion raises the in‐cylinder wall temperature, resulting in an elevated EGT.^[^ ^62^ ^]^ At maximum workload, the EGT for BDf20 is 6.45% higher than that of a pure diesel fuel. This increase is due to the higher oxygen concentration of biodiesel, which increases combustion efficiency.^[^ ^60^ ^]^

H_2_ induction has a considerable impact on EGT. This is due to the fast flame speed, enhanced combustion characteristics, and broad combustibility spectrum. The rapid transmission of the H_2_ flame intensifies the combustion cycle, resulting in greater in‐cylinder wall temperatures and thus a rise in EGT compared with normal diesel and biodiesel ignition. The increase in EGT at 12 L min^−1^ is primarily due to rapid heat release from H_2_’s high flame speed and the presence of localized high temperature zones.^[^ ^65^ ^]^ However, excessive H_2_ displaces a larger fraction of intake air, reducing the overall oxygen availability and promoting mixture stratification with locally over‐lean and over‐rich zones.^[^ ^66^ ^]^ These conditions lead to incomplete combustion of the biodiesel component, increased wall heat losses, and delayed oxidation that continues into the exhaust stroke.^[^ ^67^ ^]^ As a result, more thermal energy is lost with the exhaust gases rather than getting converted into useful mechanical work, thereby causing BTE to drop despite an increase in EGT. The EGT of BDf20 + H_2_ (12 L min^−1^) at 1.26, 2.2, 3.14, 4.08, and 5.02 kW was 171.68, 206.1, 251.48, 286.1, and 300.06 °C, respectively. These values were found to be 32.37, 19.29, 16.51, 16.52, and 11.42% higher than those of diesel fuel, with EGT values of 129.7, 172.77, 215.84, 245.54, and 269.31 °C, respectively. Compared to BDf20 + H_2_ (10 L min^−1^), the growth rates were 1.88, 2.20, 1.54, 2.36, and 3.02%, respectively. The increase in EGT for BDf20 + H_2_ (12 L min^−1^) results from the faster flame speed alongside a more extensive ignition spectrum for H_2_, resulting in an elevated burning intensity and HRR. Higher temperatures in the cylinder are caused by faster oxidation reactions alongside improved air‐fuel combinations while the H_2_ flow rate increases, leading to more thermal energy generation.^[^ ^59^, ^61^ ^]^ During increasing H_2_ flow rates, the lean mixture ignition phenomenon might occur, affecting combustion performance significantly. At maximum load of 5.02 kW, EGT for BDf20, BDf20+H_2_ (4 L min^−1^), BDf20 + H_2_ (6 L min^−1^), BDf20 + H_2_ (8 L min^−1^), BDf20 + H_2_ (10 L min^−1^), and BDf20 + H_2_ (12 L min^−1^) are 286.65, 275.25, 280.22, 287.13, 294.55 and 300.06 °C, which are found to be 6.45, 2.21, 4.06, 6.62, 9.37 and 11.42% higher than that of conventional diesel fuel (EGT of 269.31 °C), respectively. The results of Ndayishimiye et al.^[^ ^68^ ^]^ are similar to those in the current work. This supports the notion that H_2_ enrichment promotes combustion effectiveness alongside EGT, owing to H_2_’s fast flame velocity and improved ignition qualities.

### 4.2 Emission Characteristics

#### 4.2.1 Carbon Monoxide Emission

**Figure 6:**
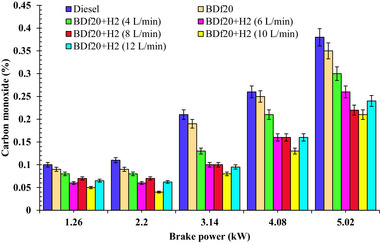
CO emission (%) with respect to engine brake power (kW) for diesel, BDf20, and BDf20 + H_2_ gas flow rate at (4, 6, 8, 10, and 12 L min^−1^).

Carbon monoxide (CO) formation occurs mainly in a fuel‐rich zone, where the air‐fuel mixture doesn't have sufficient oxygen for complete oxidation to CO_2_. The occurrence of CO in pollutants has been influenced by variables including air‐fuel ratio, ignition temperature, engine workload, and diesel blend. Elevated levels of CO pollution are typically found under rich ignition conditions and moderate in‐cylinder temperatures that prevent efficient combustion.^[^ ^60^ ^]^ **Figure** **6** shows the variance in CO output over engine workload for each of the prepared test fuel blends including diesel fuel, BDf20, BDf20 + H_2_ (4, 6, 8, 10, and 12 L min^−1^) at varying engine operating conditions of 1.26, 2.2, 3.14, 4.08 and 5.02 kW, respectively.

As the load increases, the air‐fuel ratio becomes richer, while the limited availability of oxygen hinders the entire conversion of carbon to CO_2_, which causes increased CO production. In addition, excessive fuel injection at higher loads might result in reduced combustion performance and insufficient time for complete oxidation, contributing to higher CO levels. In the case of the prepared blend BDf20, CO emissions values of 0.09, 0.10, 0.19, 0.25, and 0.35% were found to be 10, 9.09, 9.52, 3.85, and 7.89% lower than those of diesel fuel with CO values of 0.1, 0.11, 0.21, 0.26, and 0.38% at 1.26, 2.2, 3.14, 4.08 and 5.02 kW loads, respectively. This decrease is possibly due to the increased oxygen concentration of BDf20, which leads to better oxidation of carbon‐based molecules during ignition.^[^ ^59^ ^]^ The increased supply of oxygen from biodiesel facilitates more complete fuel combustion, which reduces the generation of partially oxidized carbon molecules like CO. As a result, the biodiesel's oxygen‐rich structure helps to reduce CO as compared to conventional diesel fuels.

H_2_ enrichment additionally reduces CO emissions owing to the high H_2_ diffusivity, thereby promoting superior air‐fuel rate plus combustion performance.^[^ ^61^ ^]^ The higher flame velocity and broader combustibility spectrum of H_2_ allow for improved combustion of the energy sources, reducing CO production. Furthermore, the inducement of H_2_ lowers the overall carbon footprint throughout the chamber. The reduction in hydrocarbon‐originated fossil fuel greatly reduces the required amount of carbon during partial oxidation, contributing to an overall decrease in CO emissions.^[^ ^62^ ^]^ CO emissions from BDf20 with H_2_ enrichment at 8 L min^−1^ were measured as 0.07, 0.10, 0.16, and 0.22% at load conditions of 1.26, 2.20, 3.14, 4.08, and 5.02 kW, respectively. These values represented reductions of 30.00, 36.36, 52.38, 38.46, and 42.1% compared to those from diesel fuel under the same operating conditions. The observed decrease may be due to the improved combustion characteristics provided by the H_2_ enrichment, which improves the rate of oxidation.^[^ ^42^ ^]^ Additionally, the CO associated with the BDf20 + H_2_ (10 L min^−1^) was significantly lower (over 50, 63.64, 61.90, 50, and 44.74%) as compared to diesel fuel under the same workload. This significant reduction is mainly due to the greater diffusivity and combustion efficiency of H_2_, which favours complete oxidation of fuel and reduces CO production. On the other hand, CO emissions for BDf20 + H_2_ (12 L min^−1^) at 1.26, 2.2, 3.14, 4.08, and 5.02 kW were 0.065, 0.062, 0.095, 0.16, and 0.24%, respectively, and were found to be 35.0, 43.64, 54.76, 38.46, and 36.84% lower than that of diesel fuel, respectively. These values remained inferior to diesel fuel, but somewhat higher (by a value of 30.0, 45.0, 18.75, 23.08, and 14.29%) than BDf20+ H_2_ (10 L min^−1^) at all loading conditions. Several variables contribute to the increase in CO emissions at an H_2_ flow rate of 12 L min^−1^. This trend can be attributable to the displacement of intake air by excess H_2_, which reduces the overall oxygen availability inside the combustion chamber. The resulting oxygen‐deficient regions limit the complete oxidation of CO to CO_2_, particularly in locally rich or quench‐prone zones. Additionally, the rapid heat release from H_2_ combustion can shorten the high‐temperature residence time required for complete CO oxidation, allowing more CO to escape into the exhaust. At high H_2_ flow rates, H_2_ displaces some oxygen in the inlet charge, resulting in a partial oxygen deficit during ignition. This causes the incomplete oxidation and increases CO production regardless of the H_2_ presence. Furthermore, with elevated H_2_ concentrations, the air‐fuel mixture gets richer, causing partial combustion of carbon‐based molecules and increasing CO emissions. At a maximum load of 5.02 kW, CO emissions for BDf20, BDf20 + H_2_ (4 L min^−1^), BDf20 + H_2_ (6 L min^−1^), BDf20 + H_2_ (8 L min^−1^), BDf20 + H_2_ (10 L min^−1^), and BDf20+H_2_ (12 L min^−1^) were 0.35, 0.3, 0.26, 0.22, 0.21 and 0.24%, respectively, which are found to be 7.89, 21.05, 31.58, 42.11, 44.74 and 36.84% lower than that of diesel fuel (CO emission of 0.38%), respectively. The results of the present investigation are congruent with those published by Rahman et al.^[^ ^69^ ^]^ who show a comparable pattern of CO emission reduction using H_2_ enrichment in diesel‐biodiesel blends.

#### 4.2.2 Carbon Dioxide Emission

**Figure 7:**
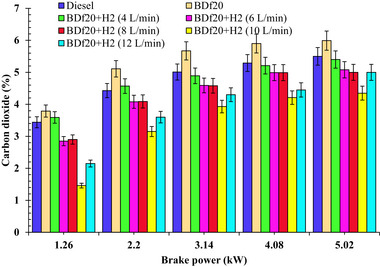
CO_2_ emission (%) with respect to engine brake power (kW) for diesel, BDf20, and BDf20 + H_2_ gas flow rate at (4, 6, 8, 10, and 12 L min^−1^).

Carbon dioxide (CO_2_) is produced by the complete combustion of hydrocarbon‐based energy sources when carbon atoms combine with oxygen molecules in an environment with sufficient oxygen availability. CO_2_ generation implies the complete combustion of fuels instead of a partial combustion, which produces CO. The amount of CO_2_ produced depends on the carbon content of the fuel and its combustibility. CO_2_ emissions are inversely correlated with CO and unburned hydrocarbon (HC) pollutants. This shows that increasing CO_2_ output results in lower CO and HC emissions.^[^ ^60^ ^]^ A better air‐fuel mixture and greater in‐cylinder wall temperature improve the combustion rate, resulting in increased CO_2_ production and reduced CO and HC pollutants. **Figure** **7** shows how distinct testing fuels affect CO_2_ emissions under various workload situations.

As shown in Figure 7, the BDf20 blend has the highest CO_2_ emissions across all workload conditions. The incorporation of additional oxygen in biodiesel improves the combustion process, allowing for the complete combustion of carbon‐containing molecules.^[^ ^61^ ^]^ Furthermore, an increase in in‐cylinder wall temperatures accelerates the transformation of partially burned‐out products to produce CO_2_.^[^ ^62^ ^]^ BDf20 with CO_2_ emission values of 3.79, 5.11, 5.67, 5.9, and 5.99% were found to be 10.17, 15.36, 13.15, 11.54, and 8.91% higher than that of diesel fuel with CO_2_ values of 3.44, 4.43, 5.01, 5.29, and 5.5% under 1.26, 2.2, 3.14, 4.08, and 5.02 kW workloads, respectively. The increase may be due to the increased oxygen concentration of biodiesel, which improves its combustibility, resulting in faster combustion of carbon‐based molecules.

The use of H_2_ reduces CO_2_ emissions by partially replacing carbon‐based fuels with an environmentally friendly, carbon‐neutral alternative. This replacement reduces the percentage of carbon throughout the combustion process, reducing CO_2_ emissions and improving performance.^[^ ^59^ ^]^ Due to the carbon‐neutral nature of H_2_, some energy sources are eliminated whenever H_2_ is introduced into the combustion process, reducing the overall carbon concentration in diesel fuel blends. Furthermore, the addition of H_2_ increases the rate of combustion, resulting in an improved oxidation phase. Ordinary diesel fuel and biodiesel combustion produce higher concentrations of CO_2_ in the exhaust than H_2_‐enriched biodiesel. This means that HC and CO have been transformed into CO_2_ in the presence of H_2_ enrichment.^[^ ^42^ ^]^ It could be seen that the CO_2_ emission from BDf20 + H_2_ (8 L min^−1^) decreased by 15.70, 7.67, 8.58, 5.67, and 9.09% respectively, compared to conventional diesel fuel at 1.26, 2.2, 3.14, 4.08 and 5.02 kW workload. Such a reasonable reduction can be attributed to the higher combustion efficiency, resulting in the complete oxidation of CO and HC. In addition, BDf20 + H_2_ (10 L min^−1^) reduces CO_2_ emissions by about 57.56, 28.89, 21.76, 20.42, and 20.91%, depending upon the workload. This reduction is mostly attributed to the more substantial substitution of carbon‐based petroleum using H_2_, thereby contributing to decreased carbon accessibility towards combustion, thereby reducing CO_2_ generation from the exhaust emissions. For BDf20 + H_2_ (12 L min^−1^), CO_2_ emissions increase to an approximate value of 13.34% compared to BDf20+ H_2_ (10 L min^−1^) due to higher combustion temperatures, enhanced oxidation of carbon‐based fuel, and the H_2_’s role in improving combustion efficiency across different loading conditions. At the higher load of 5.02 kW, the higher in‐cylinder pressure and temperature promote complete ignition, resulting in increased CO_2_ emissions. At 12 L min^−1^ of enriched induction, H_2_ works as an ignition booster, resulting in a more complete oxidation of the carbon‐based fuel. Effective combustion results in higher CO_2_ generation due to faster energy decomposition. At high H_2_ flow rates, flame instability, along with local over‐leaning, could contribute to misfire zones that could partially counteract the CO_2_ output. However, the primary impact of excessive loads involves enhanced carbon oxidation, resulting in maximum CO_2_ emissions at 12 L min^−1^ of H_2_ induction. At peak load of 5.02 kW, CO_2_ emissions for BDf20 + H_2_ (4 L min^−1^), BDf20 + H_2_ (6 L min^−1^), BDf20 + H_2_ (8 L min^−1^), BDf20 + H_2_ (10 L min^−1^), and BDf20 + H_2_ (12 L min^−1^) are found to be 5.99, 5.4, 5.08, 5.0, 4.35 and 5.02% respectively, which are 8.91, 1.82, 7.64, 9.09, 20.91 and 8.73% compared to diesel fuel (CO_2_ emission of 5.5%), respectively. These results are similar to those of Akar et al.^[^ ^70^ ^]^

#### 4.2.3 Unburnt Hydrocarbon Emission

**Figure 8:**
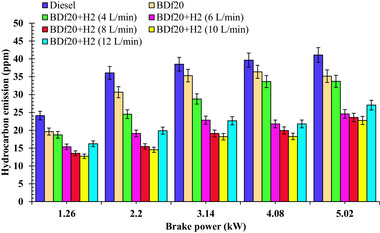
HC emission (ppm) with respect to engine brake power (kW) for diesel, BDf20, and BDf20 + H_2_ gas flow rate at (4 L, 6, 8, 10, and 12 L min^−1^).

Unburnt hydrocarbon (HC) emissions result from the partial combustion of the fuel‐air mixture inside the combustion chamber. This happens because the fuel source does not oxidise sufficiently, accumulating residual hydrocarbons in the exhaust gases. Unburnt HC is formed as a result of improper air‐fuel mixing, a low ignition temperature, inadequate oxygen supply, and flame dampening along the chamber sidewalls. The amount of HC emitted is determined by engine workload, fuel characteristics, injector settings, and combustion chamber conditions.^[^ ^42^ ^]^ **Figure** **8** shows the change in HC emissions for the tested, comprising diesel fuel, BDf20, BDf20+H_2_ (4, 6, 8, 10, and 12 L min^−1^) over changing workloads of 1.26, 2.2, 3.14, 4.08, and 5.02 kW, respectively.

It has been observed that as the engine load increases, more fuel is delivered to the combustion zone, resulting in partial ignition and a subsequent increase in HC emissions from the fuel‐rich regions. Nevertheless, compared with diesel fuel, the BDf20 blend produces fewer HC emissions. This reduction may be due to the oxygen‐rich molecular configuration of biodiesel, which improves combustibility by increasing fuel oxidation.^[^ ^62^ ^]^ The presence of intrinsic oxygen in biodiesel promotes better ignition, reduces HC emissions, and improves the overall combustion system. The HC emissions for the BDf20 blend were 19.63, 30.67, 35.31, 36.38, and 35.16 ppm, which were found to be 18.62, 14.96, 8.32, 8.24, and 14.45% lower than those of diesel fuel with HC emissions of 24.12, 36.07, 38.51, 39.65, and 41.1 ppm at 1.26, 2.2, 3.14, 4.08, and 5.02 kW workloads, respectively. This decrease is due to the oxygenated nature of biodiesel, which improves the combustion process.

Figure 8 also shows that H_2_ introduction reduces the release of HC by increasing diffusivity and accelerating flame propagation, thereby improving combustion properties.^[^ ^60^ ^]^ The H_2_ inclusion reduces diesel fuel consumption and facilitates the complete oxygenation of 8‐hydrocarbons‐derived diesel fuel, greatly reducing residual fuel remnants in the emissions. BDf20 + H_2_ (8 L min^−1^) reduced HC emissions by 36.25, 57.1, 50.39, 49.71, and 41.47% compared to diesel fuel at 1.26, 2.2, 3.14, 4.08, and 5.02 kW loads, respectively. Likewise, BDf20 + H_2_ (10 L min^−1^) reduced HC emissions by 47.30, 59.69, 52.77, 53.95, and 42.65% respectively, under the same load conditions. The considerable decline in HC emissions, due to H_2_ enrichment, is attributed to improved combustion efficiency, increased flame speed, and better oxidative properties of H_2_, resulting in an improved and more efficient phase of combustion, including lesser unburnt hydrocarbon residues.^[^ ^61^ ^]^ The inclusion of BDf20 + H_2_ (12 L min^−1^) results in a subtle deviation from the usual decreasing pattern. At high loads, the high flame velocity of the H_2_ should theoretically increase combustion efficiency, but at 12 L min^−1^, it causes unburned fuel to escape, resulting in lean‐burn misfire zones. Excessive H_2_ enrichment can induce uneven ignition scheduling, with some sections of the blend igniting rapidly and others experiencing flame extinction because of quenching effects near the cylinder walls. Higher H_2_ enrichment might alter the local equivalence ratio, producing over‐lean and fuel‐rich pockets that impede the complete combustion of the biodiesel pilot spray. Fuel droplets evaporate into zones with poor turbulence, leading to partial burning and increased hydrocarbon formation. These actions cause partially used fuel to accumulate in the exhaust, thereby increasing HC emissions. Furthermore, the shorter residence period of the charge at high flame rates does not allow for the full oxidation of hydrocarbons. Furthermore, H_2_ enrichment above ideal levels lowers the local equivalence ratio, resulting in unburned reacted hydrocarbons dripping through the engine exhaust. At a peak load of 5.02 kW, the HC emissions for BDf20, BDf20+H_2_ (4, 6, 8, 10, and 12 L min^−1^) are found to be 35.16, 33.71, 24.58, 23.59, 22.75 and 27.05 ppm, respectively, which are 14.45, 17.97, 40.19, 41.47, 42.65 and 34.18% lower than that of diesel fuel with HC emission of 41.1 ppm. In the literature, Rocha et al.^[^ ^53^ ^]^ carried out a similar study, and their results are in line with those obtained in the current research, confirming the influence of H_2_ enrichment on improving combustion performance and reducing hydrocarbon emissions in diesel engines.

#### 4.2.4 Oxide of Nitrogen Emission

**Figure 9:**
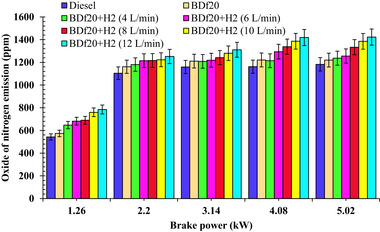
NOx emissions (ppm) with respect to engine brake power (kW) for diesel, BDf20, and BDf20 + H_2_ gas flow rate at (4, 6, 8, 10, and 12 L min^−1^).

Nitrogen oxides (NO_x_) are formed when nitrogen in the air entering the engine reacts with oxygen in the combustion chamber at elevated temperatures. The key factors in NO_x_ formation are the increase in cylinder wall temperature, oxygen accessibility, and dwell time during maximum ignition temperature.^[^ ^63^ ^]^ **Figure** **9** depicts the variation in NO_x_ emissions for the different fuels studied, including diesel fuel, BDf20, BDf20+H_2_ (4, 6,8,10, and 12 L min^−1^), under all load conditions of 1.26, 2.2, 3.14, 4.08, and 5.02 kW, respectively.

As the engine load increases, the in‐cylinder temperature increases, resulting in higher NO_x_ emissions.^[^ ^59^, ^64^ ^]^ As seen in Figure 9, the NO_x_ emissions generated by the BDf20 were determined to be 573.87, 1161.33, 1210.72, 1221.11, and 1220.07 ppm for 1.26, 2.2, 3.14, 4.08 and 5.02 kW of engine load, which are 5.64, 5.12, 4.42, 5.09, and 3.15% higher than the conventional diesel fuel having NO_x_ emissions of 543.27, 1104.74, 1159.33, 1161.93, and 1182.72 ppm, respectively. This increase in NO_x_ emissions can be attributed to the improved oxygen concentration of BDf20, which enhances combustion performance in addition to increasing the cylinder temperature.^[^ ^60^, ^68^ ^]^

H_2_ enrichment increases NO_x_ pollution because it has a much higher calorific heating value (about two times) than that of biodiesel, leading to an increase in cylinder wall temperature. Moreover, the superior combustibility properties of H_2_ lead to an improved and faster ignition, increasing its peak temperatures, while stimulating NO_x_ generation.^[^ ^62^, ^69^ ^]^ In general, the H_2_ induction with BDf20 blend increases the in‐cylinder wall temperature, triggering an increase in NO_x_. This effect occurs as the high energy density and fast burning properties of H_2_, which increases the HRR and peak cylinder temperature. Increased temperatures accelerate the chemical reaction involving nitrogen and oxygen in the combustible air, resulting in the generation of NO_x_.^[^ ^53^ ^]^ NO_x_ emissions for BDf20 + H_2_ (10 L min^−1^) have been determined to be 29.31, 17.27, 10.41, 19.27, and 17.10 more than that of the diesel fuel during 1.26, 2.2, 3.14, 4.08, and 5.02 kW. In comparison, the NO_x_ emissions of BDf20 + H_2_ (12 L min^−1^) were found to range about 44.40, 13.31, 13.03, 22.11, and 20.27%, which are greater than that of the emissions from diesel fuel over the equivalent workload extents. Higher NO_x_ emissions at increased loads are mostly associated with enhanced combustion performance and higher combustion temperature as a result of H_2_ induction. NO_x_ emissions were observed to increase with higher H_2_ enrichment, reaching a peak of 1422.4 ppm at 12 L min^−1^ of H_2_ at full load (5.02 kW), with a 2.69% increase over BDf20 + H_2_ (10 L min^−1^) and 20.27% over diesel fuel. This is because of enhanced combustion temperatures, faster flame propagation, and elevated in‐cylinder pressure owing to the high reactivity of H_2_, which together promote thermal NO_x_ formation.^[^ ^42^ ^]^ The increase in NOx at 12 L min^−1^ might be attributable to the formation of localized high‐temperature zones despite overall oxygen dilution. The flame speed and broader flammability range of H_2_ can cause advanced combustion phasing and rapid heat release, which elevates peak in‐cylinder temperatures early in the cycle. These localized temperature spikes enhance thermal NOx formation.

Although H_2_ displaces some of the intake air, leading to a slight reduction in oxygen concentration, the dominant thermal effect of H_2_ combustion, especially at full load, facilitates greater thermal NO_x_ formation. Thus, the increase in NO_x_ emissions with H_2_‐enriched induction is mainly due to the increased combustion temperature.^[^ ^61^, ^70^ ^]^ At peak load of 5.02 kW, NO_x_ emissions for BDf20, BDf20 + H_2_ (4 L min^−1^), BDf20 + H_2_ (6 L min^−1^), BDf20 + H_2_ (8 L min^−1^), BDf20 + H_2_ (10 L min^−1^), and BDf20 + H_2_ (12 L min^−1^) are 1220.07, 1236.71, 1255.5, 1333.49, 1384.84 and 1422.4 ppm, respectively, which are found to be 3.16, 4.56, 6.15, 12.73, 17.10 and 20.27% lower than that of diesel fuel with NO_x_ emission of 1182.72 ppm. These results are consistent with the previous studies by Khatri et al.^[^ ^71^ ^]^ who reported increased NO_x_ in dual‐fuel engines using H_2_‐enriched fuel blends. While H_2_ enrichment improves combustion and engine efficiency, it requires strategies such as EGR or after‐treatment technologies to mitigate the associated increase in NO_x_ emissions.

### 4.3 Combustion Behavior

#### 4.3.1 Ignition Delay Period

**Figure 10:**
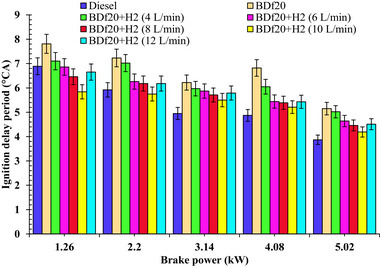
IDP (°CA) variation with respect to engine brake power (kW) for diesel, BDf20, and BDf20 + H_2_ gas flow rate at (4 L, 6, 8, and 10 L min^−1^).

The ignition delay period (IDP) is the time between the start of fuel injection and the start of combustion in the combustion chamber. The duration of IDP is crucial for understanding ignition properties, since it affects engine performance, efficiency, and greenhouse gases. Numerous variables contribute to IDP, notably fuel properties, in‐cylinder wall temperature, fuel‐air mixture, and atmospheric conditions. A shorter IDP often results in better ignition and reduces unburned fuel accumulation, whereas a longer IDP could result in increased greenhouse gas pressure rates.^[^ ^72^, ^73^ ^]^ **Figure** **10** depicts the variation of IDP with respect to engine workload across the different test fuels investigated, including diesel fuel, BDf20, BDf20+H_2_ (4, 6, 8, 10, and 12 L min^−1^).

The results displayed in Figure 10 indicate that the IDP decreases with increasing brake power. This is primarily due to the increase in in‐cylinder temperatures at higher engine loads, which increases the reactivity of the fuel‐air mixture and reduces the IDP. Enhanced atomization and better mixing at peak load also contribute to a shorter IDP, promoting improved combustion efficiency.^[^ ^74^ ^]^ The IDP of BDf20 is determined by two main variables, such as elevated oxygen concentration and inadequate mixture characteristics. The higher oxygen content in BDf20 facilitates faster oxidation, resulting in a shorter IDP. However, its poor mixability, due to increased kinematic viscosity, prevents adequate vaporization as well as air‐fuel interface, which can prolong the IDP. These competing effects create complex ignition characteristics in the IDP of the prepared blends.^[^ ^75^ ^]^ At peak load (5.02 kW), BDf20 exhibited an IDP of 5.15 °CA, ≈33.07% longer than conventional diesel (3.87 °CA). This prolonged IDP is primarily attributed to the inadequate mixing and vaporization properties of BDf20, which override the benefits of its oxygen‐rich nature.^[^ ^76^ ^]^

Enriching BDf20 with H_2_ reduces the IDP compared to BDf20 because the prepared H_2_‐air mixture takes less time to ignite. Despite its high auto‐ignition temperature, the superior diffusivity, short quenching distance, and increased flame propagation speed of H_2_ compensate for this limitation by accelerating the premixed phase of combustion. As a result, H_2_ enrichment improves early ignition and reduces IDP as flow rates increase.^[^ ^28^, ^77^ ^]^ At a peak load of 5.02 kW, the IDP for BDf20 + H_2_ (10 L min^−1^) is 4.19 °CA, which is 18.64, 16.53, 9.70, and 6.05% lower than that for BDf20 (5.15 °CA), BDf20 + H_2_ (4 L min^−1^) (5.02 °CA), BDf20 + H_2_ (6 L min^−1^) (4.64 °CA), and BDf20 + H_2_ (8 L min^−1^) (4.46 °CA), respectively. This consistent decrease in IDP with increasing H_2_ flow rate highlights the effectiveness of H_2_ in increasing the reactivity of the air‐fuel mixture and promoting an earlier onset of combustion,^[^ ^13^, ^59^ ^]^

Following an additional increase in H_2_ flow rate to 12 L min^−1^, the IDP increased slightly across all workloads. At a 5.02 kW load, BDf20+H_2_ (12 L min^−1^) showed an IDP of 4.36 °CA, somewhat higher than that at 10 L min^−1^ (4.19 °CA). This increase in delay could be ascribed to the extra H_2_, which causes over‐leaning of the mixture, reducing the combustibility of the load and thus prolonging the onset of ignition. The same trend is observed in other workloads. This indicates that at higher specific levels of H_2_ enrichment, the benefits of faster combustion with H_2_ tend to be saturated or reversed due to dispersion alongside inadequate energy density, resulting in a small increase in IDP. At a maximum load of 5.02 kW, the IDP for BDf20, BDf20+H_2_ (4 L min^−1^), BDf20 + H_2_ (6 L min^−1^), BDf20 + H_2_ (8 L min^−1^), BDf20 + H_2_ (10 L min^−1^), and BDf20 + H_2_ (12 L min^−1^) was 5.15, 5.02, 4.64, 4.46, 4.19 and 4.51 °CA, respectively, which are found to be 33.07, 29.72, 19.90, 15.25, 8.26 and 16.53% higher than that of diesel fuel (IDP of 3.87 °CA). The results from this investigation are consistent with previous research published by Kowthaman et al.^[^ ^78^ ^]^ who also found similar trends under comparable test conditions.

#### 4.3.2 In‐Cylinder Pressure

**Figure 11:**
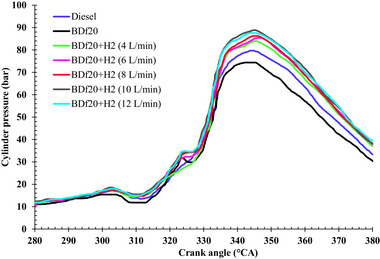
CP (bar) variation with respect to crank angle (°CA) for diesel, BDf20, and BDf20 + H_2_ gas flow rate at (4, 6, 8, 10, and 12 L min^−1^).

In‐cylinder pressure (CP) is a critical indicator of combustion characteristics in a diesel engine. It is primarily influenced by factors including the amount of fuel injected during the premixing phase, the ignition delay period (IDP), the fuel‐air mixing, and flame propagation. **Figure** **11** depicts the change in cylinder pressure as a function of crank angle for all the test fuels evaluated, namely diesel fuel, BDf20, BDf20+H_2_ (4, 6, 8, 10, and 12 L min^−1^).

Higher engine loads result in an increase in cylinder pressure (CP) due to a greater quantity of conventional fuel being fed into the combustion chamber, resulting in a higher combustible energy output. As the content of the fuel increases at higher loads, a larger proportion of the air‐fuel mixture is combusted, resulting in higher peak CP in the combustion chamber. Furthermore, at the premixed combustible stage, which is controlled by the IDP and fuel properties, differences in peak pressure levels and crank angle occurrences are added. At maximum workload (5.02 kW), the maximum CP of BDf20's was found to be 74.38 bar, ≈5.14% lower than diesel fuel. This decrease in maximum CP is possibly due to the increased kinematic viscosity of the blend and the lower energy density, which inhibits fuel evaporation and blend development, resulting in partial ignition.^[^ ^72^ ^]^ In addition, the increased residual heat from the vaporization of BDf20 produces a cooling effect within the cylinder, reducing the rapid increase in CP associated with normal diesel combustion. The high viscosity of BDf20 extends the duration of the IDP by degrading conventional fuel atomization as well as air‐fuel mixture, allowing for the ignition of a greater amount of energy sources. Furthermore, the higher latent heat of evaporation of BDf20 improves heat release from the combustion chamber, contributing to a longer IDP. As a result of the extended IDP, a significant amount of test fuel accumulates in the combustion chamber prior to the start of the combustion process, resulting in an extremely intense premix combustion.^[^ ^74^ ^]^

H_2_ addition to biodiesel blends has a significant effect on combustion characteristics. H_2_ has a higher caloric value and diffuses rapidly, resulting in faster and more complete combustion.^[^ ^76^ ^]^ Under full‐load conditions, the H_2_ addition to biodiesel blends increases the maximum CP. This is evident from Figure 11, where the BDf20 + H_2_ (10 L min^−1^) has the highest peak CP, followed by BDf20 + H_2_ at 8, 6, and 4 L min^−1^, respectively. CP tends to increase with increasing H_2_ flow rate. This improvement can be attributed to the higher calorific value of H_2_, the faster flame velocity, and the ability to form a homogeneous charge alongside fresh air, which improves the ignition performance and boosts the rate of pressure rise throughout the premix phase.^[^ ^77^ ^]^ For BDf20 + H_2_ (12 L min^−1^), the CP remains elevated compared to diesel fuel and the lower H_2_ flow blends, demonstrating that H_2_ enrichment continues to provide positive effects. However, BDf20 + H_2_ (12 L min^−1^) exhibits an in‐cylinder pressure of 87.47 bar, which shows a slight decrease of ≈1.07% when contrasted with BDf20 + H_2_ (10 L min^−1^), indicating the onset of a rich mixture condition where abundant H_2_ begins to replace incoming air, reducing the amount of oxygen required for complete combustion of the BDf20 element. This causes localized over‐fueling and marginal partial combustion, resulting in a lower pressure rise considering the higher energy input. Such conditions also lower charge density and may affect flame front behavior, resulting in ignition instability and pressure plateauing.^[^ ^79^ ^]^

Among the H_2_‐enriched fuel samples, BDf20 + H_2_ (10 L min^−1^) obtained a maximum CP of ≈88.42 bar, exceeding the value of diesel fuel along with all other blends studied, showing improved combustion characteristics. Such advances resulted from the synergistic effects of improved combustion characteristics, higher energy density, and superior fuel‐air mixture caused by H_2_‐enriched BDf20 blends, which reduced kinematic viscosity. With higher H_2_ induction, more of the fuel underwent rapid combustion throughout the premixed ignition phase, resulting in a faster HRR along with increased CPs.^[^ ^71^, ^72^ ^]^ At maximum load conditions, BDf20 + H_2_ (10 L min^−1^) has a maximum CP of 88.42 bar, 9.91% higher than diesel fuel. This trend in CP could be related to the greater combustibility characteristics of the H_2_‐enriched fuel, all of which result in more effective and comprehensive combustion operation. The results of the present research are consistent with those of Saravanan et al.^[^ ^80^ ^]^ revealing an analogous pattern in both combustion behavior as well as efficiency measurements.

#### 4.3.3 Heat Release Rate

**Figure 12:**
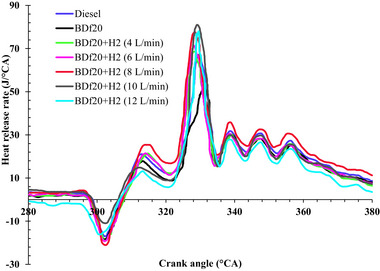
HRR (J/°CA) variation with respect to crank angle (°CA) for diesel, BDf20 and BDf20 + H_2_ gas flow rate at (4, 6, 8, 10, and 12 L min^−1^).

The heat release rate (HRR) is an important indicator that reflects the ignition and combustion characteristics within the engine cylinder. It represents the net energy released during the combustion process as a function of crank angle and provides critical insights into the magnitude, timing, and duration of the combustion event. HRR is directly influenced by fuel properties, air‐fuel mixture quality, IDP, and in‐cylinder thermodynamic conditions. A higher HRR indicates rapid energy release and contributes to improved thermal efficiency; however, excessive HRR can induce combustion instabilities, such as knocking. Understanding HRR is essential for optimizing combustion performance, controlling emissions, and improving fuel utilization.^[^ ^72^, ^74^ ^]^ **Figure** **12** illustrates the variation of HRR as a function of crank angle for different fuels tested, comprising diesel fuel, BDf20, BDf20 + H_2_ (4, 6, 8, 10, and 12 L min^−1^) according to the different loads.

At maximum engine load, the increased fuel injection enhances the energy availability within the combustion chamber, thereby improving the ignition phase. The increased HRR observed under these conditions can be attributed to increased CP and temperature, which facilitate better air‐fuel mixing and accelerate oxidation reactions. This behavior intensifies the premixed combustion phase, producing a higher peak HRR.^[^ ^76^ ^]^ Under full load conditions, BDf20 exhibits a maximum HRR of 49.2 J/°CA, ≈19.05% lower than diesel fuel. This is attributed to the LHV of BDf20, resulting in a lower energy content per unit of mass of fuel. Moreover, the higher kinematic viscosity of BDf20 adversely affects fuel atomization and vaporization, thereby weakening fuel‐air mixing and causing ignition delay.^[^ ^77^ ^]^ The higher latent heat of evaporation associated with BDf20 also leads to greater heat absorption during vaporization, reducing in‐cylinder temperature and further delaying ignition. The higher kinematic viscosity of BDf20 leads to larger droplets that persist longer in the cylinder, hindering the complete ignition and further diminishing the HRR.^[^ ^81^, ^82^ ^]^ When operating at peak load, the rise in in‐cylinder temperature improves atomization and fuel droplet break‐up. This results in better vaporization and more homogeneous fuel‐air mixtures, improving the ignition quality and combustion efficiency. The improved combustion dynamics under these conditions lead to faster oxidation of the premixed charge and a significant rise in HRR. These findings align with previously reported research outcomes.^[^ ^83^, ^84^ ^]^

At full engine load, H_2_ induction improves combustion performance by promoting better fuel‐air mixing and increasing peak HRR. The higher diffusivity and energy density of H_2_ increase the cylinder wall temperature, facilitating complete combustion, and increasing HRR.^[^ ^28^ ^]^ In the case of BDf20 + H_2_ (12 L min^−1^), the HRR is significantly higher compared to the combination of BDf20; it is marginally lower by ≈3.81% than the maximum HRR achieved with the BDf20 + H_2_ (10 L min^−1^). This slight loss in HRR with BDf20 + H_2_ (12 L min^−1^) could be due to the high H_2_ concentrations, which result in rich combustible hotspots and an inadequate air‐fuel stoichiometry. The over‐enrichment of the charge may limit the supply of oxygen required for the complete combustion of the BDf20 element, resulting in partial oxidation and a small decrease in peak HRR for increased energy input. In addition, high H_2_ flow rates may enhance thermal dilution phenomena as well as combustion vibration, affecting the overall heat release characteristics.^[^ ^47^ ^]^ At peak workload conditions, BDf20 + H_2_ (10 L min^−1^) offers a maximum HRR of 80.98 J/°CA, which is 20.82% higher than diesel fuel (HRR of 67.02 J/°CA). This increase in HRR is possibly due to the increased ignition properties enabled by the H_2_‐enriched gaseous fuel induction, such as higher calorific value as well as greater diffusion properties, which contribute to improved mixing, faster ignition, and efficient heat release. The results obtained in this study are consistent with the findings of Dimitriou et al.^[^ ^85^ ^]^ and further validate the impact of H_2_ enrichment on combustion characteristics and heat release rate in diesel engines.

## 5 Conclusion and Future Research

This research focused on the combustion, performance, and emissions of a CRDi engine fueled primarily by a Mahua biodiesel/diesel blend (BDf20) enriched with H_2_. Key findings included:

1. The BSEC decreases with increasing engine workload owing to increased combustion efficiency, although BDf20 has a slightly higher BSEC than standard diesel. H_2_ enrichment significantly reduces the BSEC, with BDf20 + H_2_ (10 L min^−1^) showing the lowest BSEC, attaining 15.58 kJ kWh^−1^, which was observed to be 10.83 and 6.13% lower than diesel fuel at a peak load of 5.02 kW, demonstrating the potential for improved energy efficiency.
2. The BTE of BDf20 was lower than that of diesel because of its higher viscosity and lower energy content, resulting in partial combustion. However, H_2_ enrichment improved BTE, with BDf20 + H_2_ (10 L min^−1^) showing the maximum improvement in the efficiency of 16.75% increase over diesel fuel, owing to the improved combustion characteristics of H_2_, including rapid flame speed and enhanced premixed combustion.
3. BDf20 had a higher EGT than diesel owing to its oxygen concentration, while H_2_ enrichment further increased it, especially at higher workloads. The maximum EGT was reported for BDf20 + H_2_ (12 L min^−1^), followed by BDf20 + H_2_ (10 L min^−1^), with an increase of about 11.42 and 9.37% compared to diesel fuel at 5.02 kW, indicating faster combustion.
4. Emissions research has shown that the oxygenated structure of BDf20 reduces CO and HC emissions compared to diesel. The addition of H_2_ significantly reduced CO and HC emissions, with BDf20 + H_2_ (10 L min^−1^) achieving a maximum reduction of 44.74% and 42.65%, respectively. Initially, BDf20 increased CO_2_ emissions, which were offset by H_2_ enrichment in its carbon‐neutral ignition. However, NO_x_ emissions increased with BDf20 and continued to increase with H_2_ enrichment owing to higher combustion temperatures and oxygen availability.
5. BDf20 had a lower HRR than diesel fuel, although H_2_ enrichment increased the HRR, especially at higher engine workloads. BDf20 + H_2_ (10 L min^−1^) had a significant HRR gain of 20.82% over diesel fuel, indicating better combustibility and reactivity properties of the fuel mixture. The CP for BDf20 was lower, but increased with H_2_ enrichment. BDf20 + H_2_ (10 L min^−1^) achieved an improved CP of 88.42 bar, which is ≈9.91% higher than diesel fuel. The IDP steadily decreased with increasing engine load at higher H_2_ flow rates. BDf20 + H_2_ (10 L min^−1^) exhibited the lowest IDP of 4.19 °C, which was 8.26% higher than diesel fuel, reflecting the enhanced reactivity and faster combustion onset owing to the favourable ignition characteristics of H_2_.

The results indicate that an H_2_ enrichment of 10 L min^−1^ with the BDf20 blend yields the most favourable balance between performance, emissions, and combustion characteristics. Increasing the H_2_ flow rate to 12 L min^−1^ leads to reduced performance improvements and higher BSEC and EGT. Emission improvements, particularly for HC, CO, NOx, and smoke opacity, were less pronounced at the 12 L min^−1^ H_2_ flow rate. This effect is attributable to excessive displacement of intake air by H_2_, which lowers the oxygen availability, thereby causing mixture stratification with locally over‐lean or over‐rich zones. These conditions disrupt the optimal combustion phasing, increase wall heat losses, and promote delayed oxidation during the exhaust stroke, thereby elevating EGT without proportional gains in engine performance and emissions. Consequently, enrichment beyond 10 L min^−1^ is thermodynamically and environmentally less effective for the engine configuration used in the current study. Overall, BDf20 + H_2_ (10 L min^−1^) provides optimal combustion efficiency, energy consumption, and thermal performance. Above this threshold, excess H_2_ negatively affects performance due to oxygen limitations, increased heat losses, and incomplete combustion. Therefore, BDf20 + H_2_ (10 L min^−1^) offers the potential to become a sustainable diesel substitute, improving engine efficiency and reducing GHG emissions. However, increased NO_x_ emissions will remain an issue, so further improvements are required. This investigation adheres to the United Nations Sustainable Development Goals (SDGs), specifically SDG 7 (Affordable and Clean Energy) by promoting renewable energy sources, SDG 13 (Climate Action) by reducing GHG emissions, and SDG 9 (Industry, Innovation, and Infrastructure) by promoting cleaner engine technologies.

Future research should focus on overcoming problems related to NOx emissions, as well as improving the combustion properties of BDf20 and H_2_‐enriched fuel blends. Potential NOx mitigation strategies include integrating advanced exhaust after‐treatment systems such as selective catalytic reduction (SCR) and exhaust gas recirculation (EGR), as well as optimizing injection timing and H_2_ flow rates. Furthermore, investigation of the effects of different H_2_ flow rates and induction tactics on combustion dynamics could provide suggestions for achieving the best combination of efficiency, GHG, and fuel economy. In addition, further research into the long‐term endurance of engine components exposed to H_2_‐enriched ignition conditions could be beneficial in ensuring both reliability and practicality. Extending studies into new biofuel substrates with enhanced fuel qualities, including lower viscosity and higher energy content, could improve the compliance and performance of dual‐fuel engines. A full life cycle assessment and techno‐economic analysis must be carried out to determine the viability of large‐scale introduction. Prospective studies should examine how other nanoparticle additions can improve engine performance and reduce pollutants. Moreover, a comprehensive energy balance analysis was not performed to quantify losses through exhaust gases, cooling systems, and mechanical friction. Such analysis might provide deeper insight into efficiency limitations and help in designing strategies to reduce losses, thereby improving the BTE of diesel engines. In general, more developments in fuel composition, engine adaptation, and pollution mitigation techniques may be required to achieve the full potential of BDf20–H_2_ dual‐fuel technology to serve as a clean and effective replacement for conventional diesel‐powered vehicles.

## Conflict of Interest

The authors declare no conflict of interest.

## Data Availability

Research data are not shared.
